# Simultaneous downregulation of miR-21 and upregulation of miR-7 has anti-tumor efficacy

**DOI:** 10.1038/s41598-020-58072-w

**Published:** 2020-02-04

**Authors:** Deepak Bhere, Nahid Arghiani, Esther Revai Lechtich, Yizheng Yao, Sarah Alsaab, Fengfeng Bei, Maryam M. Matin, Khalid Shah

**Affiliations:** 1 Center for Stem Cell Therapeutics and Imaging (CSTI), Brigham and Women’s Hospital, Harvard Medical School, Boston, MA 02115 USA; 2 Department of Neurosurgery, Brigham and Women’s Hospital, Harvard Medical School, Boston, MA 02115 USA; 3 Department of Biology and Institute of Biotechnology, Ferdowsi University of Mashhad, Mashhad, Iran; 4 Joint Center of Excellence in Biomedicine, King Abdulaziz City for Science and Technology, Riyadh, Saudi Arabia; 5 Harvard Stem Cell Institute, Harvard University, Cambridge, MA 02138 USA

**Keywords:** Cancer therapy, Targeted therapies

## Abstract

Dysregulation of miRNA expression has been implicated in cancer. Numerous strategies have been explored to modulate miR but sub-optimal delivery and inability to concurrently target multiple pathways involved in tumor progression have limited their efficacy. In this study, we explored the potential co-modulation of upregulated miR-21 and downregulated miR-7 to enhance therapeutic outcomes in heterogenic tumor types. We first engineered lentiviral (LV) and adeno-associated viral (AAV) vectors that preferentially express anti-sense miR against miR-21(miRzip-21) and show that modulating miR-21 via miRzip extensively targets tumor cell proliferation, migration and invasion *in vitro* in a broad spectrum of cancer types and has therapeutic efficacy *in vivo*. Next, we show a significantly increased expression of caspase-mediated apoptosis by simultaneously downregulating miR-21 and upregulating miR-7 in different tumor cells. *In vivo* co-treatment with AAV-miRzip-21 and AAV-miR-7 in mice bearing malignant brain tumors resulted in significantly decreased tumor burden with a corresponding increase in survival. To our knowledge, this is the first study that demonstrates the therapeutic efficacy of simultaneously upregulating miR-7 and downregulating miR-21 and establishes a roadmap towards clinical translation of modulating miRs for various cancer types.

## Introduction

MicroRNAs (miRs) are a class of small noncoding RNA that are involved in biological processes such as proliferation, differentiation, apoptosis and development^1,2^. Several studies have shown dysregulation of miRs in human cancers as a consequence of gene amplification, aberrant expression and gain of function mutations of oncogenic miRs (oncomiRs), as well as deletion or inactivation of tumor suppressor miRs (suppressor miRs)^3^.

miR-based therapeutic strategies are promising for cancer therapy^4^. However, targeting single miR is not sufficient to elicit significant therapeutic benefits *in vivo*. miR-21 is a key oncomiR, which is expressed highly in various cancer types^5–16^ and several studies have shown over-expression miR-21 promotes proliferation, invasion, and migration by repressing expression of a range of tumor suppressor genes^5–8^. Down regulation of miR-21 induces apoptosis through targeting PI3K/AKT and JAK/STAT3 signaling pathways^7^ and has a potential to collaborate or synergistically act with other miRs and fine-tune protein output^17^. microRNA-7 (miR-7) is an intronic microRNA that resides in the first intron of the heterogeneous ribonuclear protein K gene on chromosome 9^18^ and is down regulated in different cancer types^19–25^. Recently, we have shown that forced expression of miR-7 in GBM cells results in down-regulation of EGFR and p-AKT, leading to activation of the NFkB signaling^26^. As both miR-21 and miR-7 activate different yet synergistic pathways and could be a contributing factor in tumor heterogeneity, it is essential to explore the potential of co-modulation of miR-7 and miR-21 that can target complementary pathways affecting tumor growth and development.

Two different strategies for miR-targeting therapy have been explored namely, activation or upregulation of tumor suppressor miRs and inhibiting the function of oncomiRs^4^. The strategies employed to block oncomiRs include: (1) anti-miR, (2) miR sponges, (3) miR masking, and (4) small molecule inhibitors^27^. Among these strategies anti-miRNA is widely used^27,28^. However, suboptimal delivery, poor stability, toxicity and off targets have limited their applications *in vivo*. Therefore, it is important to use effective and safe systems for successful delivery of anti-miR or miR mimics to cancer cells. Alternative delivery methods have been utilized in experimental and preclinical studies, such as liposomes, extracellular vesicles (EVs), polymer-mediated delivery systems, viral vectors, cell-based delivery systems and bacteriophage-based virus-like particles (VLPs)^28,29^. However, traditional methods have presented numerous caveats such as their transient nature of inhibition and several stoichiometric restrictions that have limited the success of the anti-miR approach. Novel expression systems such as miRzip have been developed to alleviate these issues^30^. miRzip anti-sense microRNAs are stably expressed RNAi hairpins that produce short, single-stranded anti-microRNAs that competitively bind their endogenous microRNA and inhibit its function^30^.The competitive nature of miRzip binding to its target results in the de-repression and subsequent changes in the transcripts targeted by the “zipped” microRNA. On the other hand, tumor suppressor miR can serve as therapeutic strategies classified as “miR-replacement” platforms and several studies have reported positive outcomes that are comparable to or greater than gene therapies for cancer^31^.

In this study, we first assessed the role of modulating oncogenic miR-21 via miRzip in a broad spectrum of cancer types and ultimately explored the therapeutic effects elicited by simultaneous targeting miR-21 and tumor suppressor, miR-7 in various cancer types *in vivo*.

## Results

### miRzip-21 effectively downregulates miR-21, reduces invasion of cancer cells and induces apoptosis via suppression of AKT pathway

**Figure 1:**
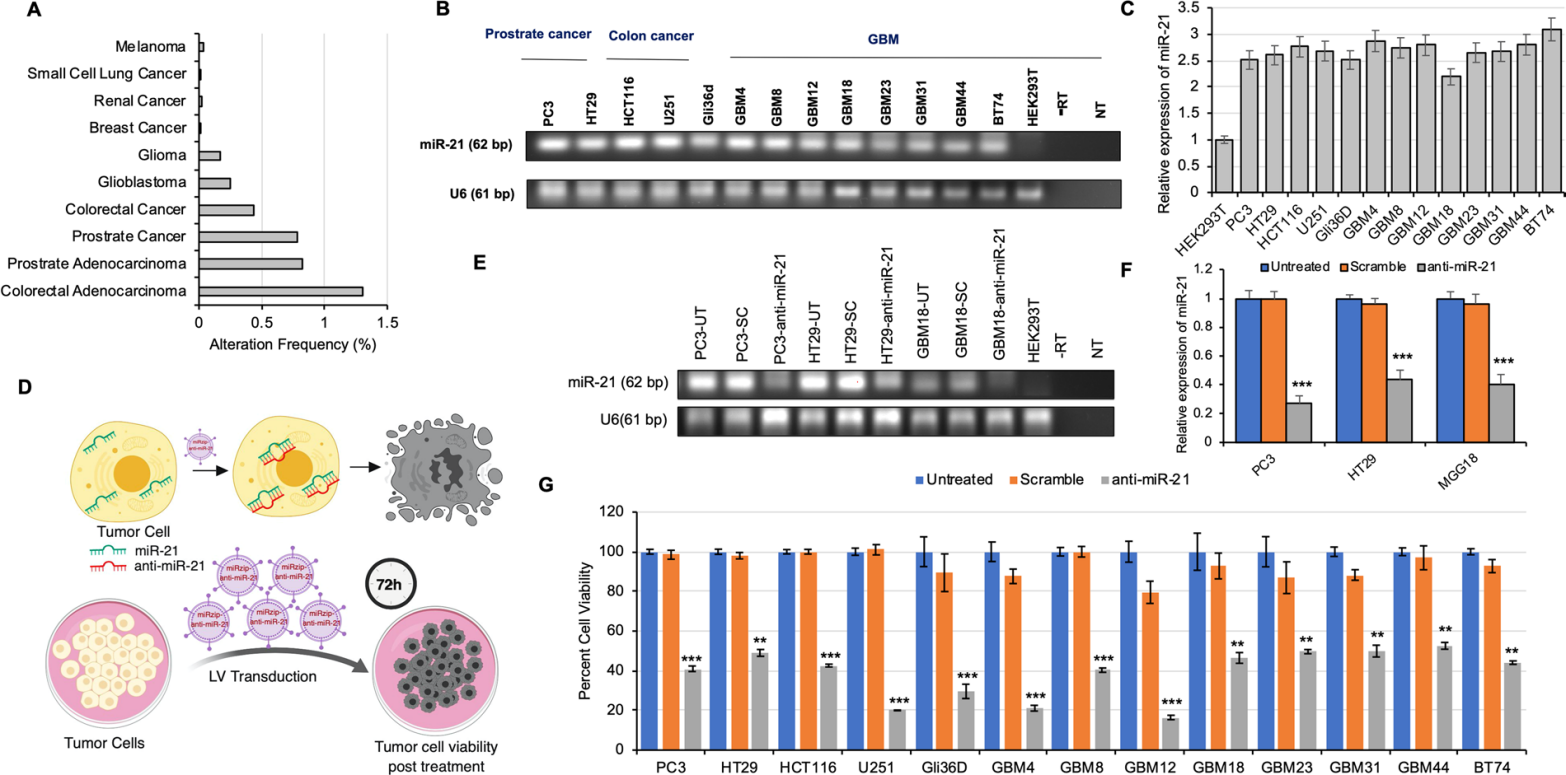
miR-21 downregulation via miRzip-21 reduces cancer cell viability *in vitro* (**A**) TCGA data showing alteration frequency of miR-21 in various cancer types. (**B**) RT-PCR analysis showing expression of miR-21 levels in various cancer types. (**C**) Plot showing relative changes in expression in miR-21 levels in tumor cells as compared to control HEK 293T cells. (**D**) Schematic representation of the experimental plan for proof-of-principle studies using the LV-miRzip-21. (E-F) RT-PCR showing changes in miR-21 levels post transduction with LVs bearing scramble miR, miRzip-21 or untreated (UT) cells. (**E**) and quantified in (**F**). (**G**) Plot showing viability of different cancer cell lines 72 h post transduction with LVs bearing scramble miR, miRzip-21 or left untreated (UT). Data are presented as mean ± SD. Significant differences between miRzip-21 transduced cells and control groups are indicated by ***(*P* < 0.001), **(*P* < 0.01) and *(*P* < 0.05).

The Cancer Genome Atlas data shows that miR-21 is up regulated in various human cancers and predominantly overexpressed in colon, prostate cancers and brain tumors (Fig. 1A). RT-PCR analysis of human prostate colon cancer and brain tumor (GBM) cell lines confirmed that miR-21 is widely overexpressed across different cancer types (Fig. 1B,C). To downregulate miR-21 in tumors cells, we packaged a lentiviral (LV) miRzip anti-sense miR-21 to stably express RNAi hairpins and investigated the efficacy of miRzip-21 mediated downregulation in different tumor cell types (Fig. 1D). RT-PCR analysis on LV-miRzip-21 transduced cells showed that miR-21 expression levels were significantly reduced as compared to control groups (Fig. 1E,F) resulting in a significant reduction in cell viability (Fig. 1G) in all cancer types including the primary patient derived GBM stem cells (GSCs). Furthermore, to understand the effect of LV-miRzip-21 following silencing of critical upstream targets, we transfected HCT116 and GBM8 tumor cells with siRNA for EGFR and AKT (Supplementary Fig. 1A,B) prior to LV-miRzip-21 treatment. siEGFR or siAKT transfected HCT116 and GBM8 cancer cells when transduced with LV-miRzip-21 showed a reduced response in cell viability as compared to wild type cells (Supplementary Fig. 1C). Morphological observations by light microscopy showed features of apoptotic cell death and reduced number of cells in miRzip-21 transduced cells as compared to controls (Supplementary Fig. 2).

**Figure 2:**
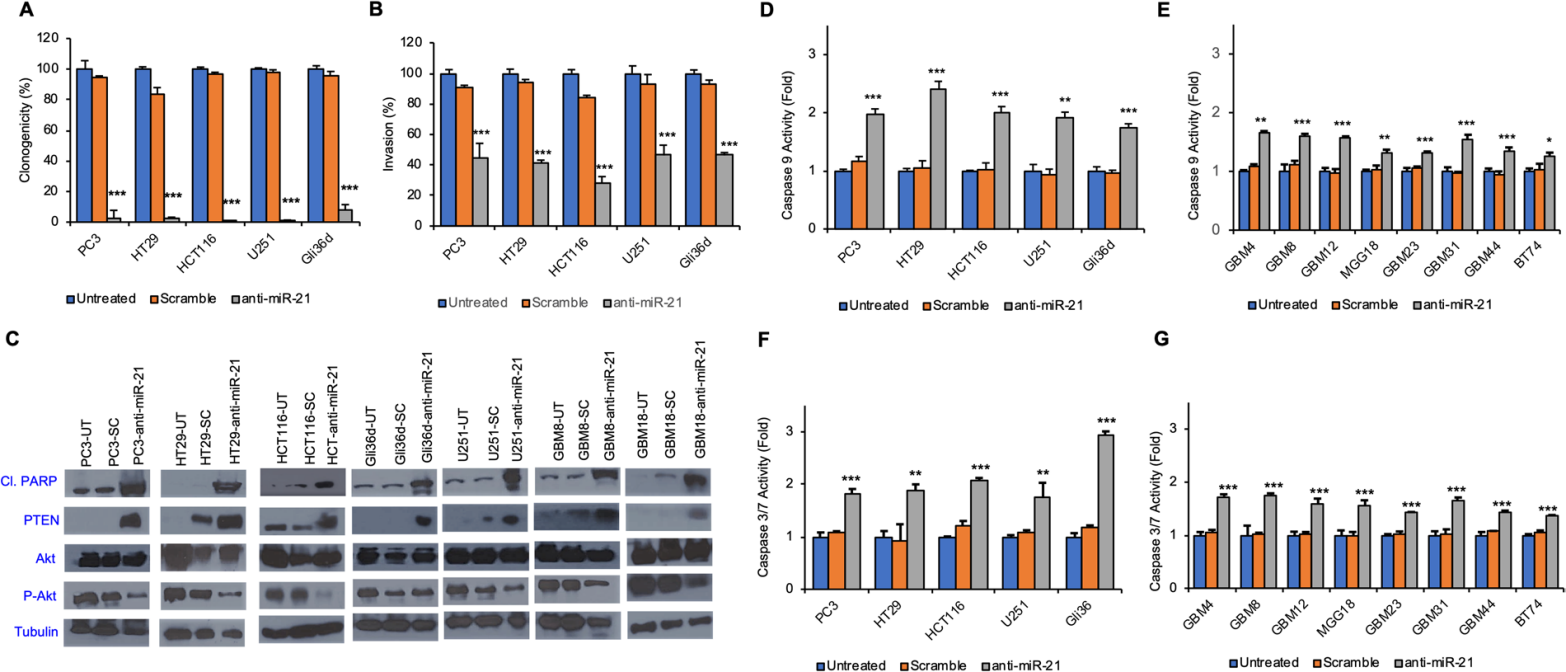
miRzip-21 results in reduced cell clonogenicity and invasion in cancer cell lines. (**A**) Plot showing changes in clonogenicity of various cancer cells following treatment with miRzip-21. (**B**) Plot showing changes in invasion of various cancer cells following treatment with miRzip-21 (**C**) Western blot analysis showing the effect of miRzip-21 in various cancer lines. (**D,E**) Plots showing changes in caspase 9 activity following treatment with miRzip-21 in various cancer cell types. (**F,G**) Plots showing changes in caspase 3/7 activity following treatment with miRzip-21 in various cancer cells. Data are shown as mean ± SD. ***(P < 0.001), **(P < 0.01) and *(P < 0.05) indicate statistical differences between miRzip-21 infected group and their relevant controls.

Next, we examined the role of miRzip-21 expression in survival, migration and invasion of tumor cells. Clonogenic assays revealed that miRzip-21 significantly inhibited the colony formation of tumor cells compared to control groups (Fig. 2A and Supplementary Fig. 3). Wound-healing assay and transwell-based cell invasion assay indicated that downregulation of miR-21 robustly inhibits the migration and invasion of cancer cells compared to scramble and untreated control cells (Fig. 2B and Supplementary Fig. 4). Western blot analyses showed that downregulation of miR-21 via miRzip-21 leads to reduced levels of activated AKT (p-AKT), a downstream target in the PTEN/AKT signaling pathway, in most cancer cells screened except Gli36d as compared to control treated cells. Moreover, the expression of PTEN, a known tumor suppressor protein, was significantly increased in tumor cells expressing miRzip-21. This resulted in tumor cells undergoing apoptosis as indicated by higher levels of cleaved-PARP (cl-PARP) and activation of caspases 3/7 and caspase 9 in miRzip-21 expressing cells compared to scramble and untreated control cells (Fig. 2C–G). A diminished caspase 9 activation was observed when miRzip-21 was -expressed in GBM8 and HCT116 tumor cells treated with siEGFR or siAKT in, the as compared to wildtype cells (Supplementary Fig. 1D). These data show that miRzip-21 effectively downregulates miR-21 and results in inducing caspase-mediated apoptosis via suppression of AKT pathway in a broad spectrum of tumor cell lines.

### AAV-miRzip-21 but not MSC-miRzip-21 attenuates cancer growth *in vitro* and *in vivo*

**Figure 3:**
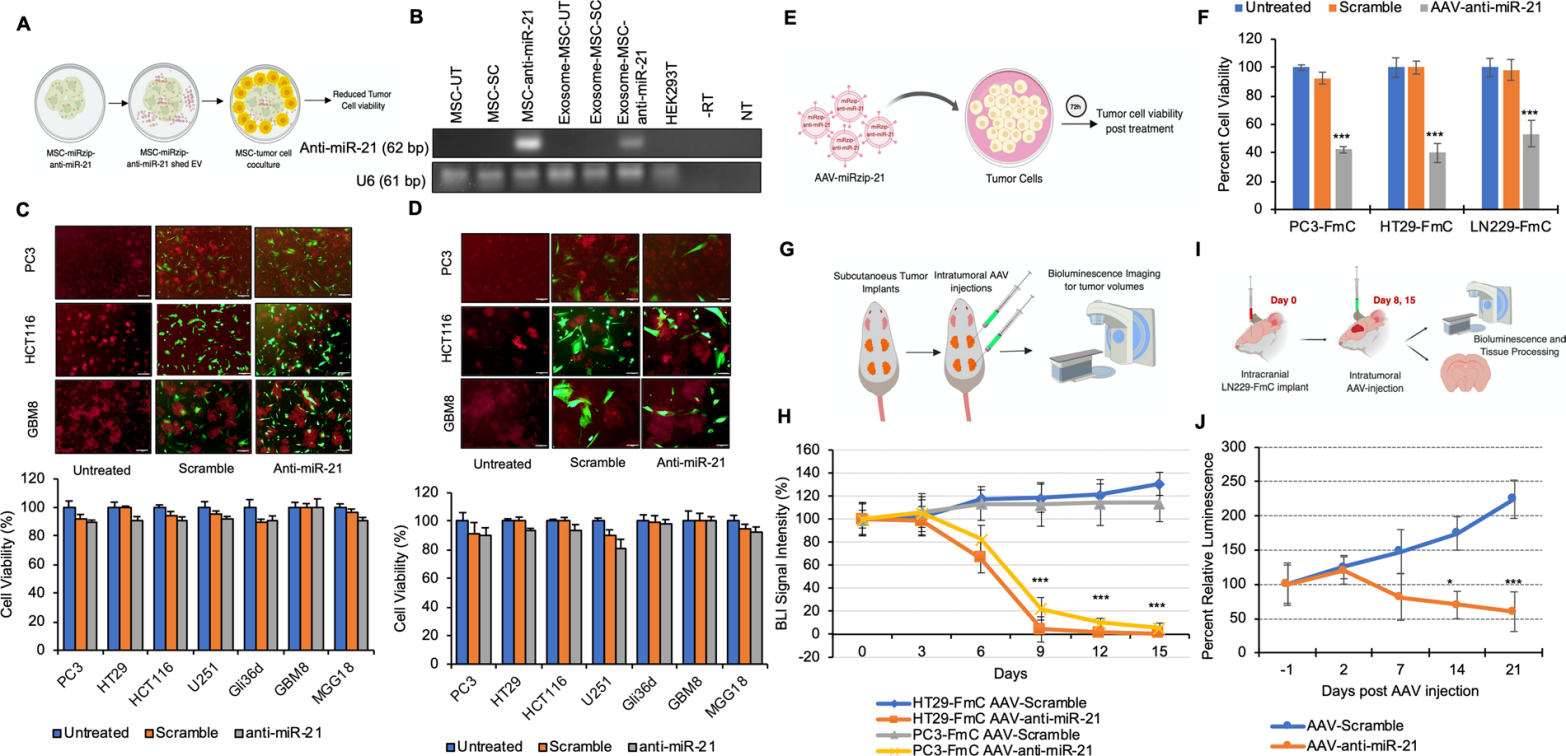
AAV-miRzip-21 effectively reduces cancer cell progression *in vivo* (**A**) Illustration showing the proposed hypothesis of MSC based delivery of anti-miR-21 to tumor cells. (**B**) RT-PCR assay showing miR-21 expression in LV-miRzip-21 transduced-MSCs and exosomes extracted from transduced MSC. Negative and RT-minus controls are indicated by NT and -RT, respectively. UT and SC represent untreated and scramble control groups, respectively. (**C**) Plots and representative fluorescent micrographs of cancer cells cocultured with LV-miRzip-21 expressing MSCs at 3:1 ratio showing cell viability at 120 h. Scale bars: 100 uM (**D**) Plots and representative fluorescent micrographs of cancer cells cocultured with LV-miRzip-21 infected-MSCs at 1:1 ratio and corresponding cell viability at 120 h. Scale bars: 50 uM (**E**) Illustration showing the model for AAV transduction of tumor cells (**F**) Plot showing changes in tumor cell viability at 72 h post transduction with AAV-miRzip-21 and control. (**G**) Illustration of the *in vivo* subcutaneous model of colon and prostate cancers. (**H**) Plot showing changes in bioluminescence signal intensity overtime following AAV-miRzip-21 injection. (**I**) Illustration of the intracranial LN229-FmC animal model. (**J**) Plot showing changes in bioluminescence signal intensity overtime following AAV injection. Data presented as mean ± SD. ***(P < 0.01) and *(P < 0.5).

Mesenchymal stem cells (MSCs) have been demonstrated to be a safe and effective delivery vehicle for miRs, due to their ability to specifically target cancer cells and to transfer molecules via exosomal trafficking^32,33^. We hypothesized that MSC-shed exosomes will serve as carriers for anti-miR-21 and these MSC can be utilized to deliver miRzip-21 to the tumors *in vivo* (Fig. 3A). To determine the exosome enrichment of anti-miR-21 from transduced MSCs, exosomes were harvested from MSCs transduced with LV-miRzip-21 and control MSC. RT-PCR analysis revealed that MSCs shed exosomes were enriched in miRzip-21 (Fig. 3B). To investigate the therapeutic efficacy of MSC-miRzip-21, different cancer cells were cocultured with MSCs at 1:1 and 3:1 ratio. No change in tumor cell viability was observed in tumor cells post-co-culture with MSC-miRzip-21 in both tested ratios (Fig. 3C,D). To further investigate enrichment of anti-miRzip-21 western blot analysis of the MSC and isolated exosomes was performed. Western blot analysis using CD63 marker to identify exosomes revealed that exosomes are enriched from MSC engineered to express anti-miRzip-21 (Supplementary Fig. 5). These data reveal that although MSC-shed exosomes are enriched in anti-miR-21, they are unable to influence tumor cell viability *in vitro*.

The delivery of transgenes via AAV provides long-term stable *in vivo* gene expression in both dividing and non-dividing cells with low risk of related genotoxicity, which makes it a useful and highly suitable option for cancer gene therapy^34–38^. AAV gene transfer technology has also shown promise in clinical trials^34,39^. To create an efficient delivery vehicle for targeting miR-21 in tumors, we created AAV bearing miRzip-21 (Fig. 3E) and tested its efficacy *in vitro*. AAV-miRzip-21 transduced cells showed a reduction in cell viability (P < 0.01) as compared to control AAV treated or untreated cells (Fig. 3F). To evaluate the efficacy of AAV-miRzip-21 *in vivo*, we first created imageable tumors lines of prostate cancer (PC3-FmC), colon cancer (HT29-FmC) and GBM (LN229-FmC) by transducing them with LVs bearing a dual fluorescent and bioluminescent marker (Fluc mCherry; FmC). Next, mice bearing flank tumors of HT29-FmC and PC3-FmC (n = 10/tumor type) were injected intratumorally with three doses of 1 × 10^6^ pfu of either AAV-scramble or AAV-miRzip-21 (n = 5/group) and mice were followed for changes in tumor volumes (Fig. 3G, H). A significant reduction in tumor volumes was seen in mice treated with AAV-miRzip-21 (P < 0.01) as compared to scramble treated groups (Fig. 3H). To evaluate the effect of AAV-miRzip-21 in intracranial GBM models, mice bearing LN229-FmC tumors were stereotactically injected intratumorally with two doses of 1 × 10^6^ pfu of either AAV-scramble or AAV-miRzip-21 (n = 5/group) (Fig. 3I,J). A significant reduction in tumor volumes (P < 0.01) was seen in mice treated with AAV-miRzip-21 as compared to scramble treated groups (Fig. 3J). These data indicate that AAV-miRzip-21 effectively targets colon, prostate and brain cancer growth. However, the progression of colon and prostate tumor growth is halted more efficient than the GBM growth.

### Co-modulation of miR-21 and miR-7 prolongs survival of mice bearing GBM xenografts derived from patient GSC

**Figure 4:**
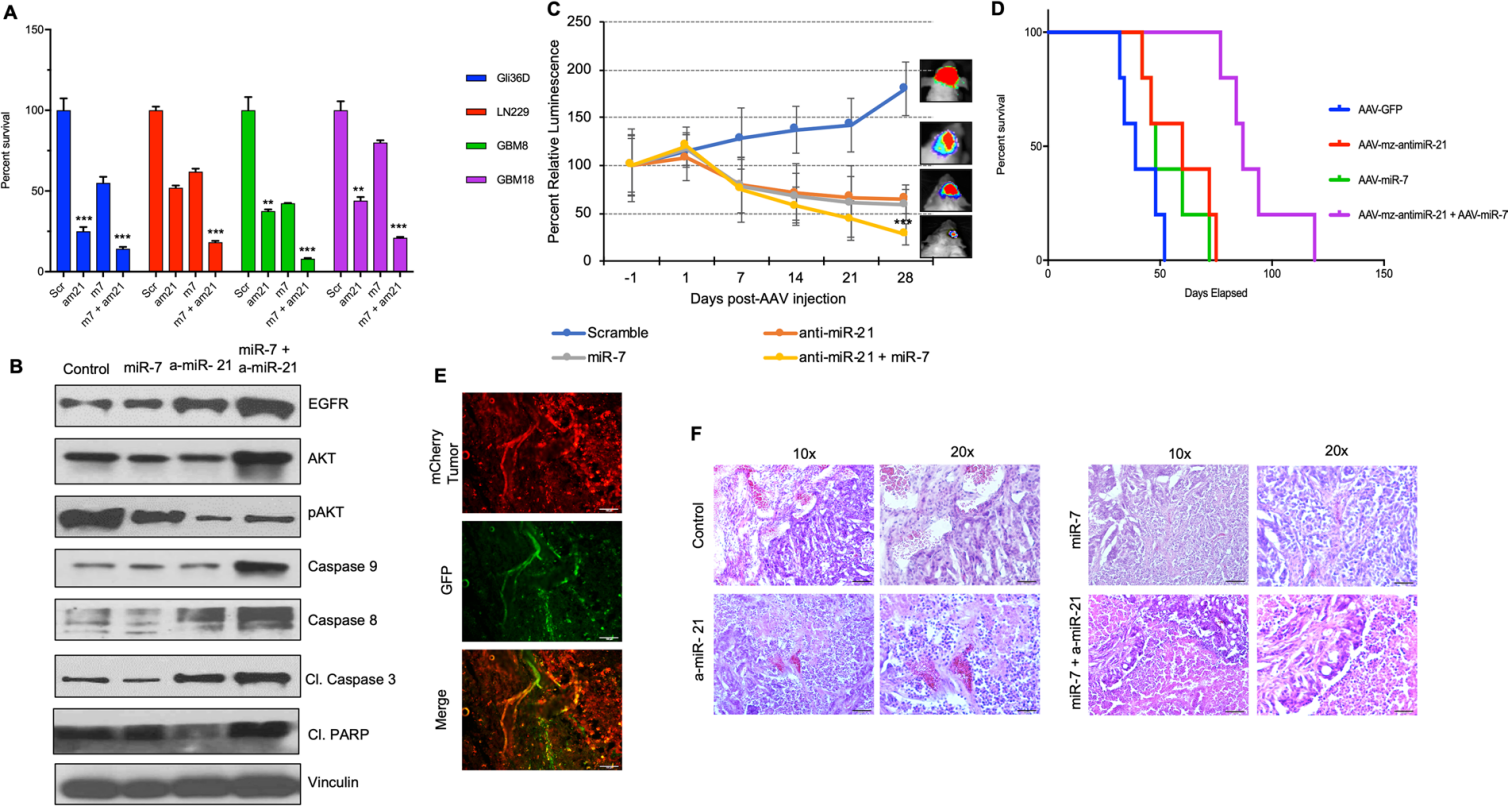
Combination of AAV delivered anti-miR-21 and miR-7 prolongs survival of mice bearing patient derived GBM xenografts. (**A**) Plot showing changes in viability of various GBM cells following combination treatment with AAV-miR-7 and AAV-miRzip-21. (**B**) Western blot analysis showing changes in various cell proliferation and death markers following treatment with AAV-miR-7 and AAV-miRzip-21. (**C**) Plot showing changes in tumor volumes of GBM-18-FmC overtime following AAV-miR-7 and AAV- miRzip-21 injections as compared to controls. (**D**) Kaplan-Meier survival curves following AAV-miR-7 and AAV-miRzip-21 injections as compared to controls. (**E**) Photomicrographs showing AAV-GFP infection of GBM tumors at day 4 post AAV injection. (**F**) H&E staining showing changes in tumor burden following a combination treatment of AAV delivered miR-7 and anti-miR-21. Data are presented as mean ± SEM. ***(P < 0.001), **(P < 0.01) and *(P < 0.05). Scale bars represent 100 uM.

We have previously shown that forced expression of miR-7 in GBM cells results in down-regulation of EGFR and p-AKT and activation of the NFkB signaling^26^. In an effort to evaluate the effect of miR co-modulation on GBM tumor progression as compared to modulation of miR-21 alone (Fig. 3J), we explored the possibility of combing the downregulation of miR-21 and upregulation of miR-7 in GBMs. We tested a cohort of established and primary patient-derived GBM stem cells (GSC) for their response to a combination of miRzip-21 and miR-7. A significant reduction in GBM cell viability (P < 0.001) was seen in all the tested GBM cell lines post-combined treatment with AAV-miR-7 and AAV-miRzip-21 treatment as compared to monotherapy and control (Fig. 4A). Western blot analysis showed that combination treatment leads to substantial changes in the proteins involved in caspase-mediated apoptosis of tumor cells, in particular, caspase-3, caspase-8 and caspase-9 cleavage and subsequent PARP cleavage (Fig. 4B). Next, we evaluated the therapeutic efficacy *in vivo* using a primary patient derived GBM model. Specifically, mice bearing patient primary GSC (GBM18) expressing a bimodal imaging marker FmC, GBM18-FmC were challenged with 1 × 10^6^ pfu of either AAV-GFP, AAV-miR-7, AAV-miRzip-21 or a combination of AAV-miR-7 and AAV-miRzip-21. A significant reduction (P < 0.001) in tumor burden was observed in mice treated with a combination of AAV-miR-7 and AAV-miRzip-21 as compared to the monotherapy and control (Fig. 4C). Kaplan Meier survival analysis showed a significant extension in survival of mice treated with the combination of AAV-miR-7 and AAV-miRzip-21 as compared to the other groups (Fig. 4D). Fluorescence imaging of brain sections revealed a robust infection of the GBM tumor following AAV injection (Fig. 4E) H&E analysis revealed a reduction in tumor burden in mice brains following dual modulation of miR-7 and miR-21 (Fig. 4F). These data reveal that modulation of miR-7 and miR-21 presents a therapeutic benefit in mice bearing GBM.

## Discussion

In this study we show that delivery of miRzip-21 effectively induced apoptosis in different cancer types *in vitro* and mouse xenografts of prostate cancer, colon cancer and brain tumors. We also demonstrate a robust improvement in therapeutic benefit in mice bearing GBM brain tumors by co-modulating miR-21 and miR-7 thereby presenting a unique approach to target heterogenic tumors.

Several studies have shown miR-21 is among the most predominantly dysregulated miR in different cancer types including prostate cancer, colon cancer and highly malignant brain tumors and there is positive correlation between miR-21 expression levels and tumor stages^5–8^. miR-21 activates the EGFR/AKT signaling pathway via targeting tumor suppressor genes such as PTEN, PDCD4, APAF1, TPM1, TIMP3, RECK, FOXO1 and SPRY2^40^. Different systems have been used to inhibit oncomiR functions such as anti-miRs, miR sponges, miR-masking and small molecule inhibitors^27^. Despite great promise of available therapeutic anti-miR systems, the effectiveness of current anti-miR strategies is hindered by their transient nature of inhibition. In this study, we used the miRzip anti-sense microRNAs which are stably expressed RNAi hairpins that have anti-microRNA activity to downregulate miR-21. miRzip systems overcome the drawbacks by providing permanent expression of short hairpin anti-sense miRNAs and have been shown to efficiently prevent recurrence of cancers without adverse effects^41^. Our findings indicate that intracellular constitutive expression of miRzip-21 has the potential to be more effective than other means of downregulating miRs in cancer cells with high level of miR-21^42^. microRNA-7 (miR-7) is an intronic microRNA that resides in the first intron of the heterogeneous ribonuclear protein K gene on chromosome 9^18^. We previously shown that forced expression of miR-7 via lentiviral vectors suppresses the EGFR/PI3K/AKT signaling pathway in GBM^26^. miR-7 is also known to induce apoptosis through inhibition of BCL2^43^ and inhibition of miR-21 activates caspase 9 and 3^7,44^.

Cancer cells activate multiple anti-apoptotic pathways to escape programmed cell death through overexpressing many of proteins. Therefore, targeting a single pathway maybe not be adequate for complete tumor control. miR-21 downregulation is known to induce apoptosis through inhibition of PI3K/AKT and JAK/STAT3^7^. Previous studies have also shown that miR-21 activates RAS/MAPK pathway through targeting antagonists of this pathway^45^ and can simultaneously inhibit PI3K/AKT and RAF/MEK/ERK in GBM^46^. miR-7 is also known to induce apoptosis through inhibition of BCL2^43^ and inhibition of miR-21 activates caspase 9 and 3^44,47^. In order to improve outcomes, simultaneously targeting both miR-21 and miR-7 pathways offers a promising strategy as they target parallel cell survival pathways and produce a synergistic effects^48,49^. Although there are few reports on forced co-expression or co-inhibition of miRNAs in cancer study^50–53^, none of the previous studies have shown simultaneous inhibition and overexpression of different miRs in the same tumor. In one study, Dong *et al*. showed co-inhibition of miR-21 and miR-10b can significantly induce apoptosis and reduce cell invasion in human GBM^50^. In an effort to improve therapeutic outcomes by miR modulation, we have explored co-modulation of miR-21 and miR-7 in this study as a strategy to target heterogenic tumors. Our findings indicate that simultaneous suppression of miR-21 and upregulation of miR-7 exert synergistic anti-cancer effects on human GBM through inhibition of EGFR and p-AKT and activation of caspase- mediated pathways, lead to prolonged survival rate of mice bearing xenografts model of patient-derived GBM^26^.

To gain insights into the molecular mechanisms underlying apoptotic effects of miRzip-21, our data showed the expression levels of cleaved-PARP, PTEN and caspase 3/7 and 9 activity were increased, while the level of p-AKT was decreased after miR-21 suppression. These results suggest that downregulation of miR-21 can increase PTEN which acts as negative regulator of PTEN/p-AKT pathway. Elevated level of PTEN results in activation of caspases 9 and 3/7. Previous studies have shown that inhibition of miR-21 in PTEN mutant cell lines suppresses activated AKT and EGFR^7^ and leads to decreased levels of STAT3 and p-STAT3^7^. In our studies, we showed miRzip-21 mediated reduced levels of activated AKT and increased level of cleaved-PARP, PTEN and caspase 3/7 and 9 activity in PTEN mutant cell lines. We have also shown that silencing critical upstream receptors and targets diminished effects of LV-miRzip-21. This validates the involvement of the EGFR/PI3K/AKT pathway and proposed mechanism of action is depicted in Supplementary Fig. 6. This is in line with the previous studies showing that downregulation of miR-21 can induce apoptosis through inhibition of EGFR in JAK/STAT3 pathway, independent of the PTEN status^29^.

Our data indicates that modulation of specific miRs such as miR-21 could be an effective therapeutic strategy for personalized cancer treatment. However, sub-optimal delivery systems have hindered their progress into clinical settings. Exosomes offer a non-toxic and non-immunogenic delivery systems, however, efficiency of their drug-loading and retention is not sufficient to have substantial therapeutic effects *in vivo*^32,33^. Previously published studies have shown that intravenously injected exosomes are rapidly cleared from the blood and accumulate in lung, liver and spleen^54,55^. Industrial scale production of exosomes is a big hurdle to overcome before exosomes are tested in the clinic for treatment of a vast array of diseases^32^. In addition, there are no published results of trials, which used exosomes as miR carriers yet^56^. Previous studies have shown that MSCs can effectively deliver miRs to cancer cells via their exosomes^57–59^. We investigated the therapeutic efficacy of miRzip-21-carrrying MSC as source of exosomes containing miRzip-21. Our results showed no significant decrease in tumor cell numbers when co-cultured with MSC-miRzip-21 probably due to insufficient amount of anti-miR-21 in exosomes derived from MSCs.

The delivery of transgenes via AAV provides long-term stable *in vivo* gene expression in both dividing and non-dividing cells with low risk of related genotoxicity, which makes it a useful and highly suitable option for cancer gene therapy^34–38^. AAV gene transfer technology has also shown promise in clinical trials^34,39^. Currently, AAV-mediated gene delivery is performed in clinical trials of several human diseases such as Parkinson’s, hemophilia A and B, cystic fibrosis, muscular dystrophy, and ocular disease^35–38^. Additionally, AAVs have been engineered to deliver anti-angiogenic, tumor suppressor, DNA repair, cytotoxic or suicide genes, cytokines, shRNAs and antibodies. These studies showed promising results in preclinical stage^4^. So far, different serotypes of AAVs including AAV1, AAV-2, AAV1-AAV2 hybrids, AAV-6, AAV-7, AAV-8, AAV-9 and AAVrh10 have been applied in more than 200 gene delivery trial studies. AAV serotype-9 (AAV9) has the ability for effective cross the blood-brain barrier (BBB) and infects CNS cells^60^. Using AAV delivered miRzip-21 was shown to have efficacy both *in vitro* and *in vivo* in a broad spectrum of tumor lines, thus offering a potential delivery system for oncomiR silencing. Among all naturally occurring AAV serotypes, AAV2 is the best characterized and extensively studied and has served as the archetype for AAV biology^36^. AAV2 serotype additionally has a broad tissue tropism as compared to the other known serotypes thus making them a choice of selection for gene therapy^36^. In this study, we have used AAV2 serotype based on our previously published studies^26^ and its efficacy in effectively delivering miR to brain tumors.

In conclusion, our data strongly reveal that AAV mediated stable silencing of miR-21 in combination with forced expression of miR-7 exerts superior anti-tumor outcomes and will serve as templates for a novel therapeutic approach in treating various cancer types.

## Materials and Methods

### Cell lines

Human Prostate (PC3) and colon (HT29 and HCT116) cancer, glioblastoma (U251 and Gli36d), human embryonic kidney transformed with large T antigen cells (HEK293T) and human mesenchymal stem cells (MSC) were utilized in this study. U251, Gli36d and HEK293T were grown in low glucose Dulbecco’s modified Eagle’s medium (LG-DMEM), HT29 and HCT116 were cultured in RPMI-1640 and McCoy’s 5 A medium (Gibco, Carlsbad, CA), respectively. All these media were supplemented with 10% fetal bovine serum (FBS, Gibco, Carlsbad, CA) and 1% penicillin/streptomycin (Invitrogen, Carlsbad, CA). Glioblastoma (GBM) stem cells (GBM4, GBM8, GBM12, GBM18, GBM23, GBM31, GBM44 and BT74) were grown in Neurobasal medium (Invitrogen, Carlsbad, CA) supplemented with 3mM L-glutamine (Invitrogen, Carlsbad, CA), B27 (Invitrogen, Carlsbad, CA), 2 µg/ml heparin (Stem Cell Technologies, Vancouver, BC), 20 ng/ml human EGF (Invitrogen, Carlsbad, CA), and 20 ng/ml human FGF-2 (Invitrogen, Carlsbad, CA) as described previously^61^. Ad-MSCs were also cultured in high glucose DMEM supplemented with 10% exosome-depleted FBS (Gibco, Carlsbad, CA) with 3 mM L-glutamine and penicillin/streptomycin (Gibco, Carlsbad, CA). All cells were incubated at 37 °C in a humidified atmosphere of 5% CO_2_ in air. Tumor cells were transduced with LV-Pico2-Fluc-mCherry (FmC) at a multiplicity of infection (MOI) of 2 in medium containing protamine sulfate (2 µg/ml). Cells were visualized by fluorescence microscopy for mCherry and selected by puromycin selection as described earlier^26^. To generate therapeutic and control MSC, MSC were transduced with LV-miRzip-21 or LV-scramble (Scr) at MOI of 5 in medium containing protamine sulfate (2 µg/ml). Cells were visualized by fluorescence microscopy for GFP expression 36 hours post transduction.

### Reverse Transcription-PCR

To determine the miR-21 levels, miRNAs were isolated from cancer cells and Ad-MSCs using miRNeasy Mini Kit (Qiagen). Stem-loop PCR was used for determining the U6, mature miR-21 and anti-miR-21 expressions. RT- PCR was performed. Reverse transcriptase reactions contained RNA samples, 50 nM stem–loop RT primer (IDT Technologies), 2x reverse transcriptase buffer (NEB, USA), 0.25 mM each of dNTPs (NEB Labs) 200 U/µl M-MLV reverse transcriptase (NEB, USA) and 40 U/µl RNase inhibitor (Thermo Fisher Scientific, USA). Total 20 µl reactions were incubated in a 384-well plate for 30 min at 16 °C, 30 min at 42 °C, and 5 min at 85 °C. PCR Reaction contained 5 µl cDNA, 25 µl Taq 2x master mix (NEB, USA), 0.5 µM of each forward and universal reverse primers. The reactions were incubated in at 95 °C for 30 sec., followed by 40 cycles of 95 °C, 55 °C for 30 s, 72 °C for 30 s and 75 °C for 5 min. No template and RT minus controls were included in each experiment.

### Cell viability assays

5 × 10^3^ cells per well were plated in 96 well plates. After 24 h, cells were infected with viruses. Cell viabilities were determined using Cell Titer-Glo Luminescent Cell Viability Assay (Promega, USA) and calculated according to the following equation for each treatment. Cell viability (%) = the mean of fluorescence in infected cells/the mean of fluorescence in control (untreated) cells × 100

### Colongenic and migration assays

1 × 10^3^ adherent cells were seeded in each well of a six well plates. 24 hours later, cells were transduced with either LV-miRzip-21 or LV-Scr. Cells were incubated for ten days, colonies were fixed and stained with 0.2% crystal violet (Sigma-Aldrich) and counted.

Wound healing assay is a widely used technique to analyze the ability of cell migration *in vitro*. Adherent cancer cells were plated in 24 well plates and then transduced with LV-miRzip-21 or LV-Scr. After 24 hours, a straight scratch was introduced using a pipette tip. Cell migration was monitored at 4 different time points and quantified using Image J1.48 v.

### Invasion assay

1 × 10^5^ LV-miRzip-21 or LV-Scr transduced cells in serum-free media were seeded on Matrigel-coated chambers of the 24 h well trans well plates (pore size 0.8 μm, Corning, USA). 700 µl of medium containing 15% FBS was used in the lower chamber. After 24 hours, cells were fixed with methanol and stained using 0.2% crystal violet. Cells were removed from upper side of the filters and stained cells on the lower side of the membranes were counted under a light microscope.

### Silencing EGFR and AKT using siRNA

HCT116 and GBM8 tumor cells were transfected with either siRNA for AKT (Thermo Fisher Scientific, Waltham, MA), siRNA for EGFR (Thermo Fisher Scientific, Waltham, MA) or scramble RNA using Lipofectamine RNAiMax reagent (Life Technologies, Carlsbad, CA) per manufacturer’s recommendations. 72 hours post transfection, cells were assayed for gene knockdown using western blotting as described below.

### Western blotting

Total protein was extracted from tumor cells following transduction with either LV-miRzip-21 or LV-Scr and quantified using Bradford protein assay. For siRNA studies, the total protein from GBM8 or HCT116 tumor cells was extracted 48 h post transfection and quantified using Bradford Assay. For exosome analysis, total protein was extracted from MSC-miRzip-21 or MSC-Scr and also from exosomes purified from culture medium by ultracentrifugation.

20 µg of the protein lysates from these samples were loaded onto 10% Mini-PROTEAN TGX Precast Protein Gels (Bio-Rad, USA) and conducted at 110 V for 75 mins. Flowing the separation step, proteins were transferred to nitrocellulose membranes at 100 V for 1 h. Membranes were blocked with 5% dry milk or 5% bovine serum albumin (BSA) and 0.1% Tween-20 in TBS for 1 h at room temperature (RT), and incubated with primary antibodies in TBST overnight at 4 °C. After treated with HRP-conjugated secondary antibodies in TBST for 1 h at RT, membranes were developed with Super Signal West Pico system (Thermo Fisher Scientific, Waltham, MA). Then, signals were visualized using autoradiographic films and quantified with Image J. We used Anti- α-Tubulin or Vinculin as a loading and internal control.

### Caspase assays

5 × 10^3^ cells/well were seeded in 96 well plates and 48 h after viral infection, caspase activities were assessed by caspase-3/7 and caspase-9 Glo reagents (Promega, USA).

### MSC Coculture experiments

Tumor cells expressing FmC and LV-miRzip-21 transduced or LV-Scr transduced -MSC were seeded at ratios of 1:1 and 3:1 in 96 well plates. Cell viabilities of the tumor cells viability were determined 96 and 144 h post coculture using bioluminescence-based Cell Viability Assay as described previously^29^.

### Adeno-associated virus packaging

Recombinant AAV was produced in HEK293T cells by standard triple transient transfection system with some modification. Briefly, three plasmids of pAAV-RC, pHelper and AAV transfer plasmid were co-transfected into HEK293T cells using polyethylenimine (PEI). Cells containing viral particles were collected 72 h after transfection, and the AAV particles were purified by iodixanol gradient (15%, 25%, 40% and 60%) ultracentrifugation, concentrated and formulated in phosphate buffered saline (PBS) containing 0.001% Pluronic F68 (Gibco). Virus titer was determined by measuring DNaseI-resistant genome copies using quantitative PCR.

### *In vivo* assessment of AAV-anti-miR-21 efficacy in colon and prostate cancer

Nude mice (3 week of age, Charles River Laboratories, Wilmington, MA) were implanted subcutaneously with HT29-FmC and PC3-FmC (1 × 10^5^ cells per flank; n = 10/group). Mice were then injected with 1 × 10^6^ pfu AAV-miRzip-21 (n = 5) or AAV-scramble (n = 5) on days 3, 5 and 7 post tumor implantations. tumor cell fate was imaged by Fluc activity as described previously^26^.

### Illustrations

All illustrations have been created by D.B. using biorender.com through an academic subscription (D.B.).

### Intracranial GBM cell implantation and *in vivo* bioluminescence imaging

To understand the effect of AAV-miRzip-21 on GBM, LN229-FmC (2.5 × 10^5^ cells per mouse, n = 10) were stereotactically implanted into the brains (right striatum, 2.5-mm lateral from bregma and 2.2-mm deep) of 8-week-old nude mice. Mice were then stereotactically injected with 1 × 10^6^ pfu AAV-miRzip-21 (n = 5) or AAV-scramble (n = 5) on days 8 and 15-post tumor implantation. GBM burden was followed in in real time by bioluminescence imaging as described previously. To assess therapeutic benefit of the combination of anti-miR-21 and miR-7 modulation, GBM18-FmC cells (5 × 10^5^ cells per mouse, n = 32) were stereo tactically implanted into the brains (right striatum, 2.5-mm lateral from bregma and 2.2-mm deep) of 8-week old nude mice. Mice were then stereotactically injected with 1 × 10^6^ pfu AAV-miRzip-21 (n = 8), 1 × 10^6^ pfu AAV-miR-7 (n = 8), 1 × 10^6^ pfu AAV-miRzip-21 and 1 × 10^6^ pfu AAV-miR-7 (n = 8) or AAV-scramble (n = 8) on days 15 and 22-post tumor implantation and followed for the GBM burden in real time by bioluminescence imaging (BLI) as described previously^26^ and also followed for survival analysis. All *in vivo* procedures were approved by the Institutional Animal Care and Use Committee at BWH. Mice demonstrating any visual signs of distress as seen by impaired locomotion or difficulty in accessing food and water were excluded from the study. Treatment groups were randomly assigned from the total number of mice that were included in the study and the investigator was not blinded while administering the treatment.

### Tissue processing, H&E staining and immunohistochemistry

GBM were prefused and brains were removed and sectioned for histological analyses. Brain sections were mounted on slides, washed in PBS and mounted and visualized for fluorescence on a confocal microscope (LSM Pascal, Zeiss, Oberkochen, Germany)^26^. For H&E staining, sections were incubated with Hematoxylin and EosinY (1% alcohol), dehydrated with 95% and 100% ethanol and mounted in xylene-based media^26^.

### Statistical analysis

For *in vitro* experiments, one-way ANOVA was used to calculate the *P* values using SPSS 16.0 software. Pair-wise comparisons were conducted using two-sample T-Test with Bonferonni adjusted P-values for the cases of multiple pair-wise tests. Data were expressed as mean ± S.E.M. or S.D. and differences were considered significant at P < 0.05. For survival analysis, Kaplan-Meier estimates and the Log-Rank test for the comparison of survival curves were conducted for all time to event outcomes.

### Supplementary information

Supplementary Information.

Supplementary Information.
