# Author Correction: Simultaneous downregulation of miR-21 and upregulation of miR-7 has anti-tumor efficacy

**DOI:** 10.1038/s41598-024-66236-1

**Published:** 2024-07-03

**Authors:** Deepak Bhere, Nahid Arghiani, Esther Revai Lechtich, Yizheng Yao, Sarah Alsaab, Fengfeng Bei, Maryam M. Matin, Khalid Shah

**Affiliations:** 1 Center for Stem Cell Therapeutics and Imaging (CSTI), Brigham and Women’s Hospital, Harvard Medical School, Boston, MA 02115 USA; 2 Department of Neurosurgery, Brigham and Women’s Hospital, Harvard Medical School, Boston, MA 02115 USA; 3 Department of Biology and Institute of Biotechnology, Ferdowsi University of Mashhad, Mashhad, Iran; 4 Joint Center of Excellence in Biomedicine, King Abdulaziz City for Science and Technology, Riyadh, Saudi Arabia; 5 Harvard Stem Cell Institute, Harvard University, Cambridge, MA 02138 USA

Correction to: *Scientific Reports* 10.1038/s41598-020-58072-w, published online 04 February 2020

The Supplementary Figure file published with the original version of this Article contains errors.

As a result of an error during the figure assembly, the anti-miR21 panel at 48 hrs got duplicated from Scramble panel at 12 hrs in Gli36d panel block of Supplementary Figure 4.

Additionally, uncropped Western blots are also provided for Figure 4B.

The correct Supplementary Figure file is now linked to this correction notice.

### Supplementary Information

Supplementary Legends.

Supplementary Figures.

