# Supplementary material for: Simultaneous downregulation of miR-21 and upregulation of miR-7 has anti-tumor efficacy
Source: Sci Rep. 2020 Feb 4;10:1779. doi: 10.1038/s41598-020-58072-w (PMC7000780; doi:10.1038/s41598-020-58072-w)
Supplement: Supplementary file 1 — Supplementary Information. [file 41598_2020_58072_MOESM1_ESM.docx]

**Simultaneous downregulation of miR-21 and upregulation of miR-7 has anti-tumor efficacy**

Deepak Bhere^1, 2,4*^, Nahid Arghiani^1, 2, 3*^, Esther Revai Lechtich^1, 2^, Yizheng Yao^2^, Sarah Alsaab^1,4^, Fengfeng Bei^2^, Maryam M. Matin^3^, Khalid Shah^1, 2, 4,5^

**Supplementary Figure Legends**

**Supplementary Fig. 1: (A)** Western blot showing modulation of EGFR and Akt in HCT116 by using siRNA. **(B)** Western blot showing modulation of EGFR and Akt in GBM8 by using siRNA. **(C)** Plot showing changes in tumor cell viability 48 h following treatment with anti-miRzip-21. **(D)** Plot showing changes in caspase-9 activity in tumor cells 48 h following treatment with anti-miRzip-21.

**Supplementary Fig. 2: (Left)** Photomicrographs of GFP fluorescence showing transduction of various tumor cells by miR scramble and miRzip-21 lentiviruses. (Right) Light microscopy photomicrographs revealing reduced number of cells in miRzip-21 transduced cells as compared to the controls

**Supplementary Fig. 3:** Light images from Clonogenic assays representing the inhibition of colony formation of tumor cells by miRzip-21 as compared to control groups.

**Supplementary Fig. 4:** Representative light images from the transwell migration assay clearly signifying a robust inhibition of tumor cells migration by miRzip-21.

**Supplementary Fig. 5:** Western blot showing CD63 expression in MSC and exosomes enriched from MSC-anti-miRzip-21.

**Supplementary Fig. 6:** Illustration depicting the proposed mechanism of action

**Supplementary Fig. 7:** Raw Western blots of all the experiments included in the main manuscript.
