# Supplementary material for: Simultaneous downregulation of miR-21 and upregulation of miR-7 has anti-tumor efficacy
Source: Sci Rep. 2020 Feb 4;10:1779. doi: 10.1038/s41598-020-58072-w (PMC7000780; doi:10.1038/s41598-020-58072-w)

Simultaneous downregulation of miR-21 and upregulation of miR-7 has anti-tumor efficacy

Deepak Bhere[1, 2,4*], Nahid Arghiani[1, 2, 3*], Esther Revai Lechtich[1, 2], Yizheng Yao[2], Sarah Alsaab[1,4],

Fengfeng Bei[2], Maryam M. Matin[3] and Khalid Shah[1, 2, 4,5]

Suppl. Figure 1

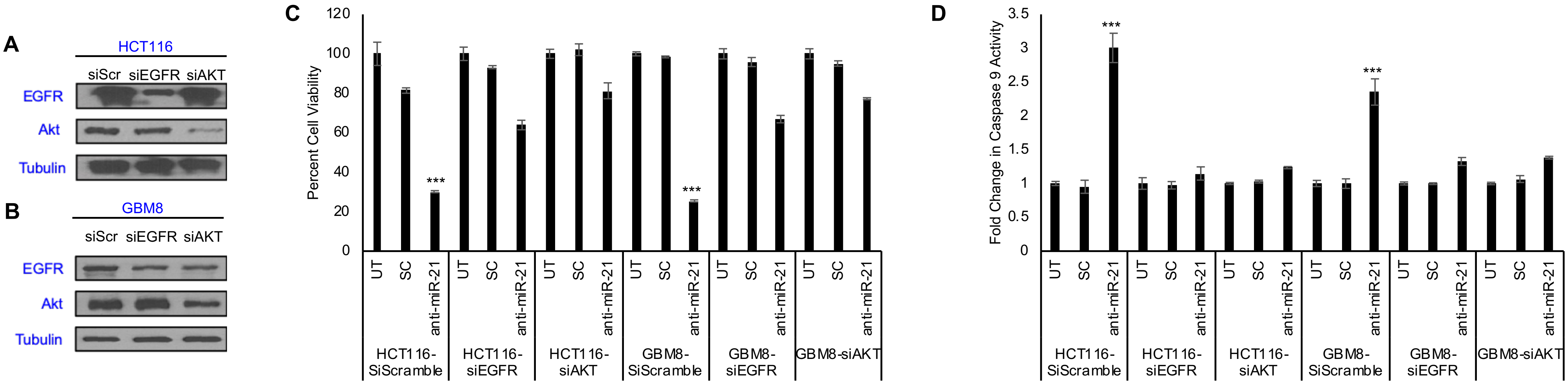

Suppl. Figure 2

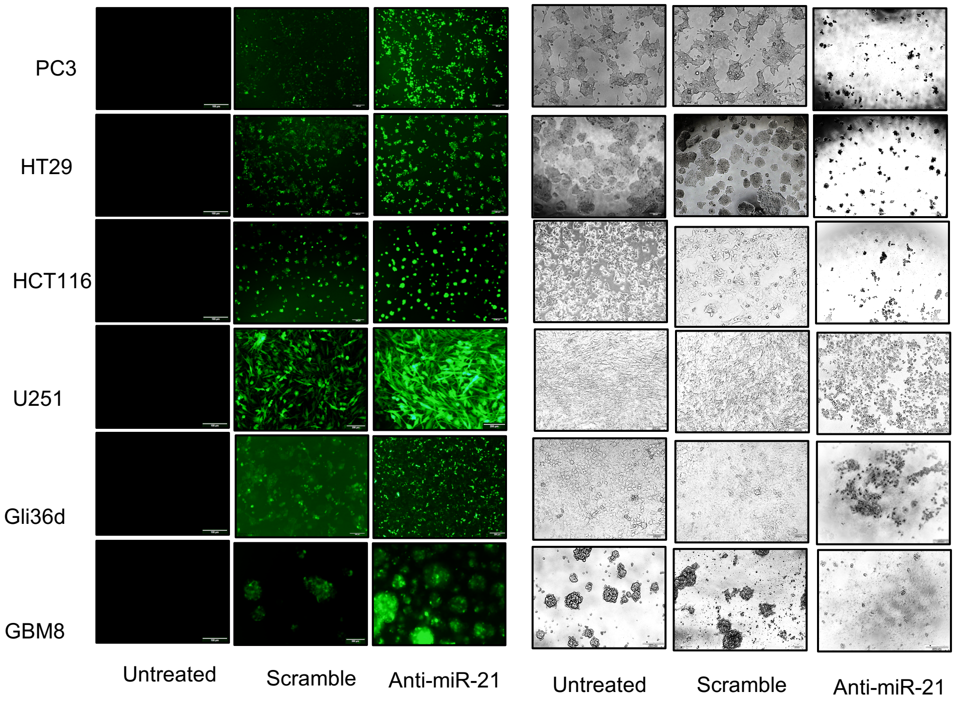

Suppl. Figure 3

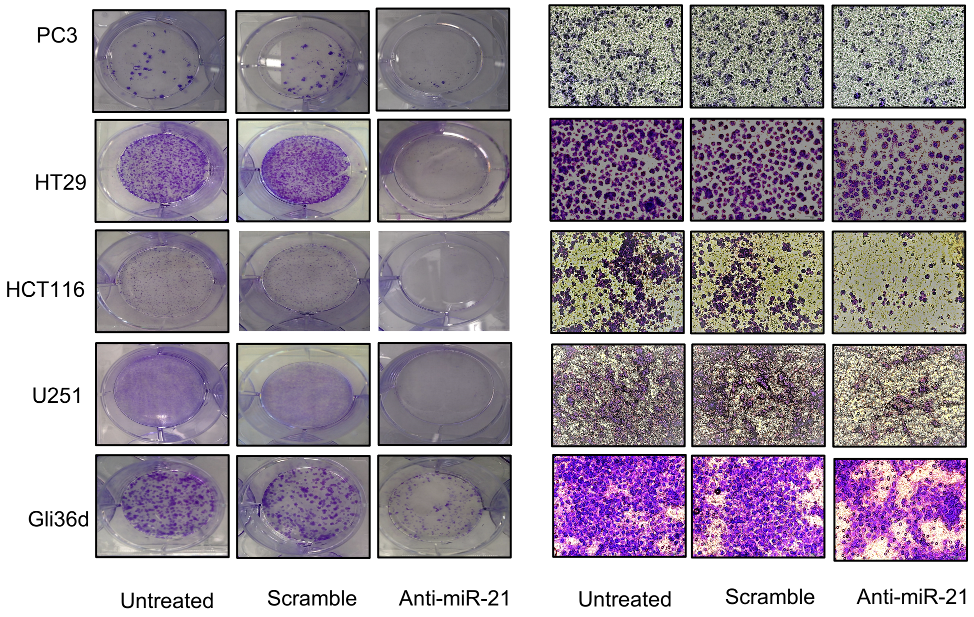

Suppl. Figure 4

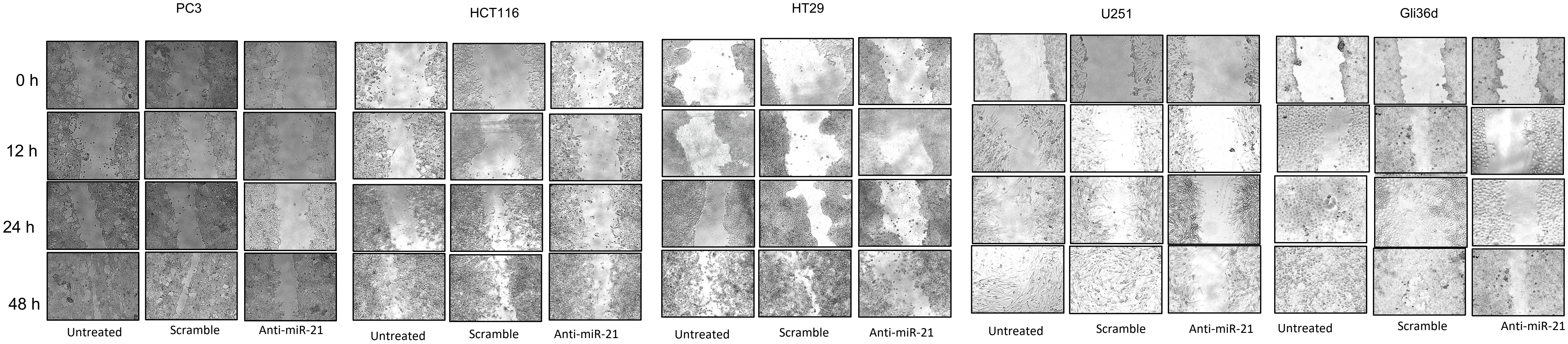

Suppl. Figure 5

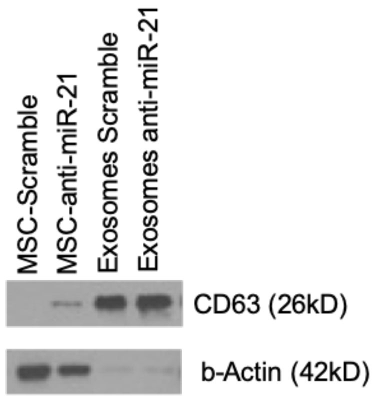

Suppl. Figure 6

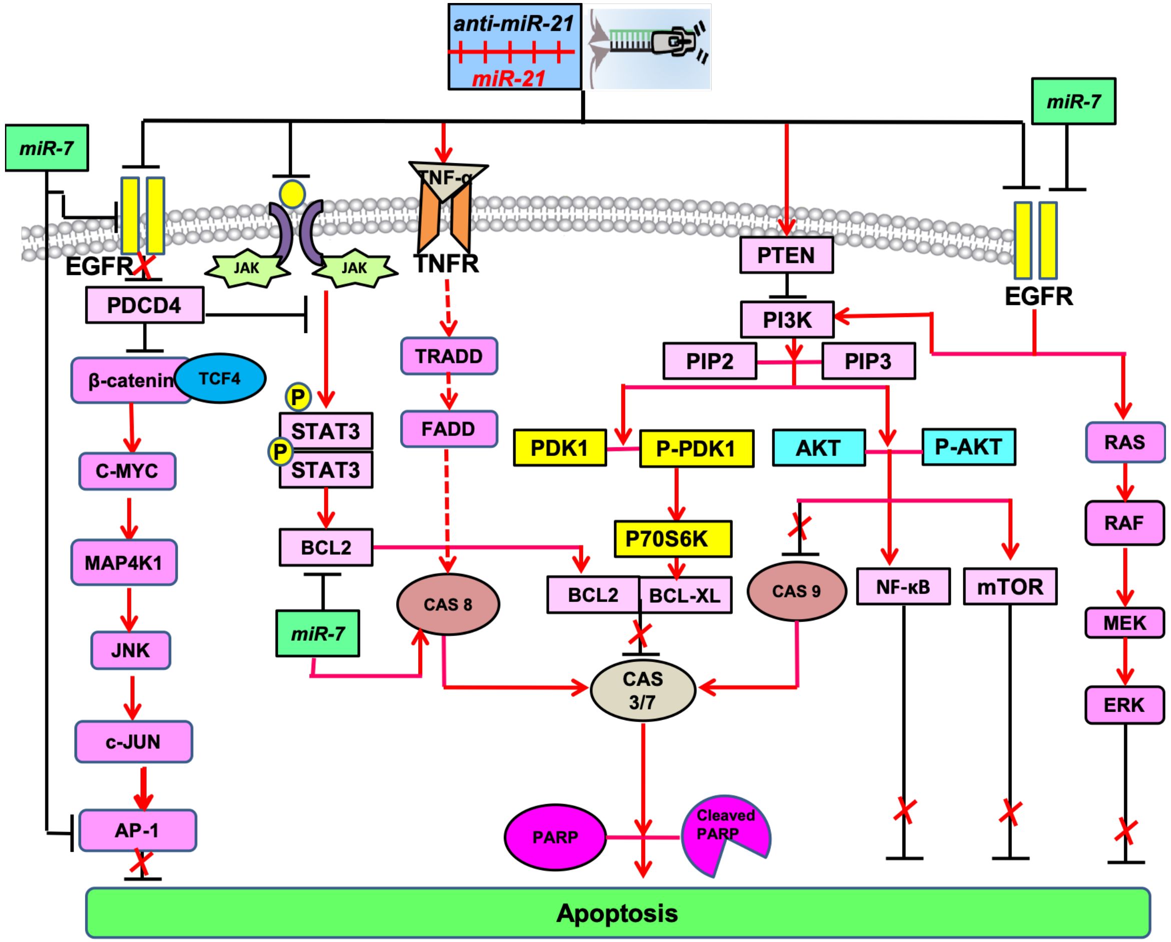

Suppl. Figure 7

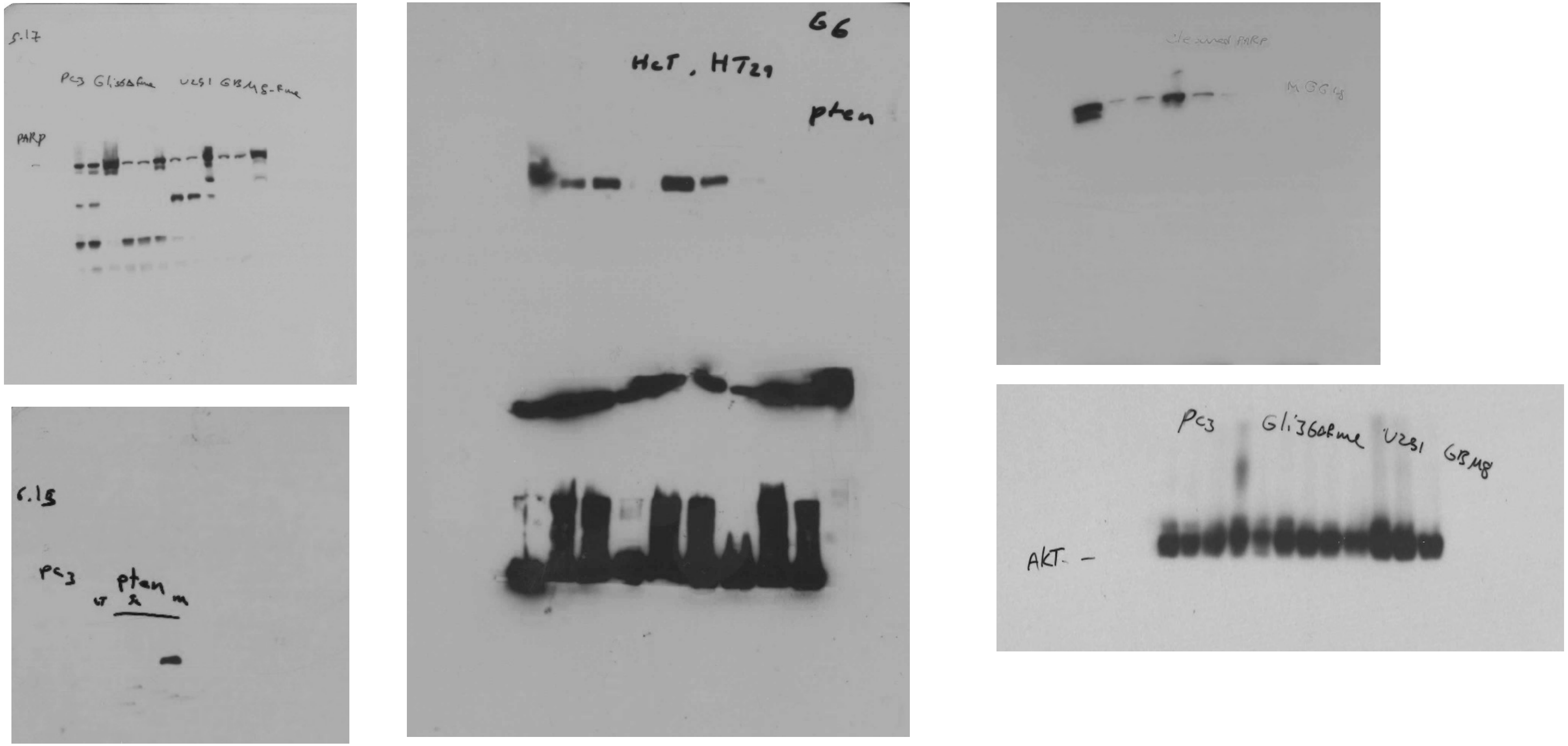

Supplement: Supplementary file 2 — Supplementary Information. [file 41598_2020_58072_MOESM2_ESM.pdf]
