# Supplementary material for: Author Correction: Simultaneous downregulation of miR-21 and upregulation of miR-7 has anti-tumor efficacy
Source: Sci Rep. 2024 Jul 3;14:15303. doi: 10.1038/s41598-024-66236-1 (PMC11222387; doi:10.1038/s41598-024-66236-1)

Simultaneous downregulation of miR-21 and upregulation of miR-7 has anti-tumor efficacy

Deepak Bhere[1, 2,4*], Nahid Arghiani[1, 2, 3*], Esther Revai Lechtich[1, 2], Yizheng Yao[2], Sarah Alsaab[1,4], Fengfeng Bei[2], Maryam M. Matin[3] and Khalid Shah[1, 2, 4,5]

Suppl. Figure 1

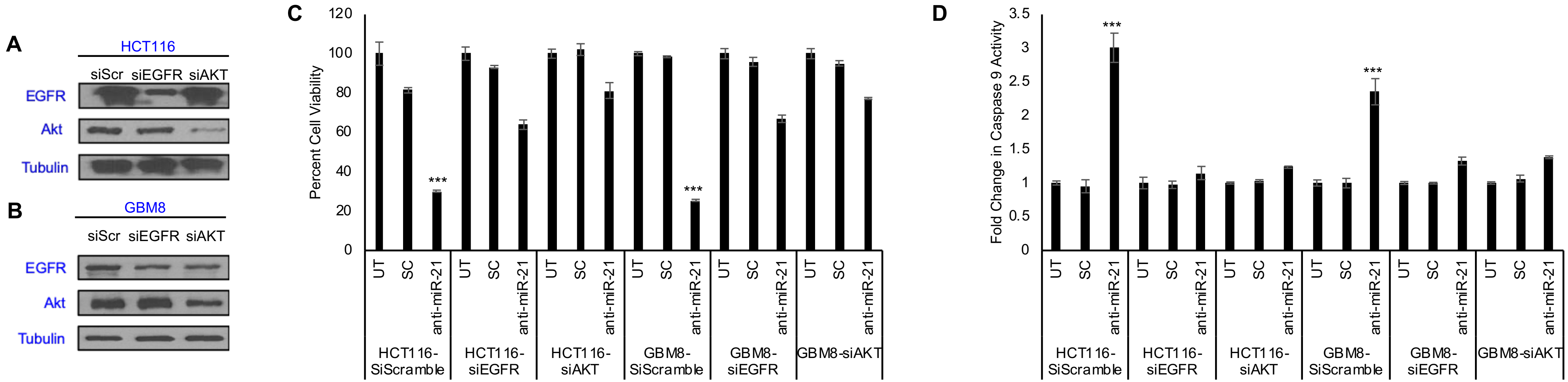

Suppl. Figure 2

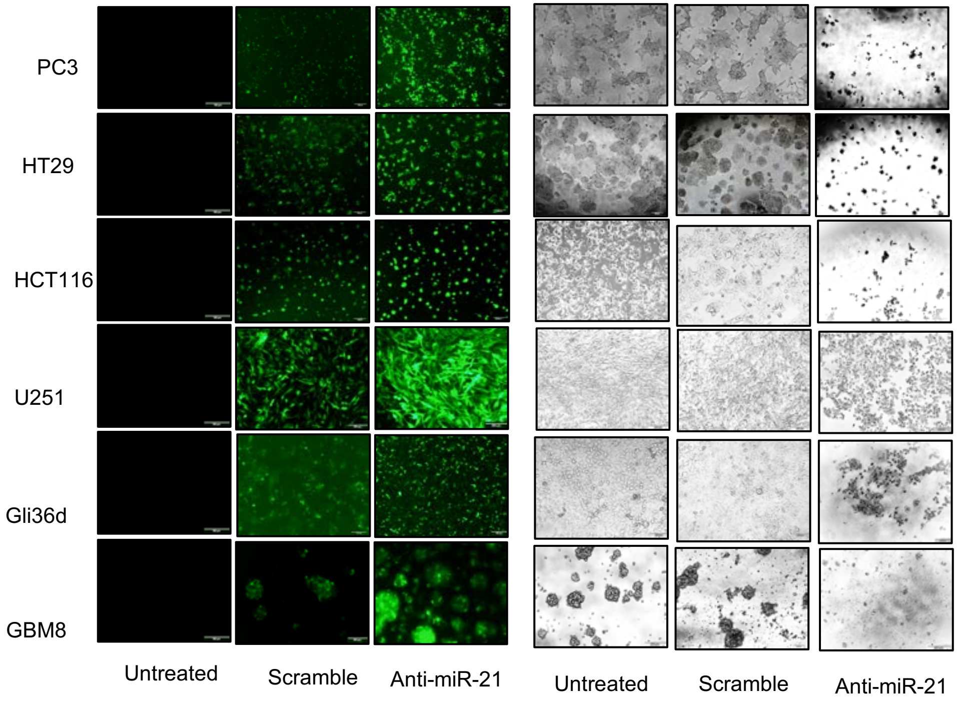

Suppl. Figure 3

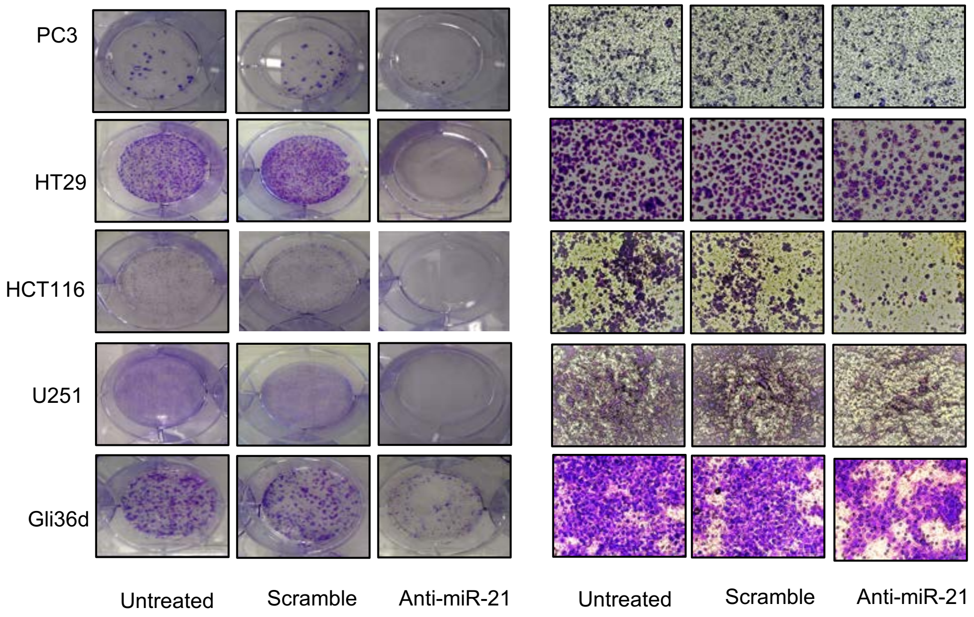

Suppl. Figure 4

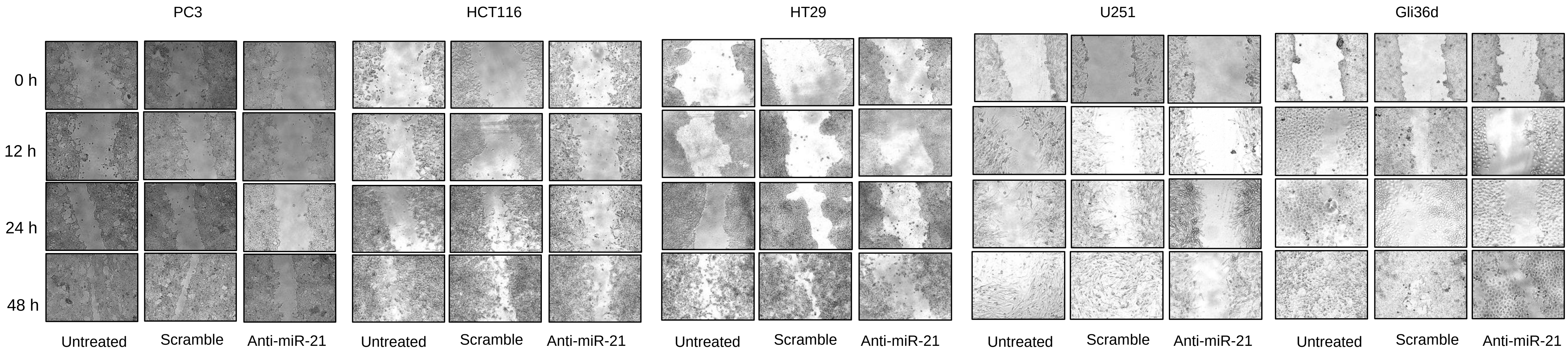

Suppl. Figure 5

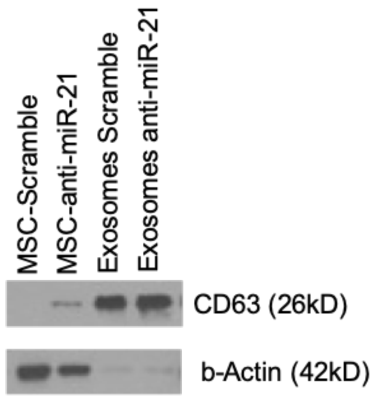

Suppl. Figure 6

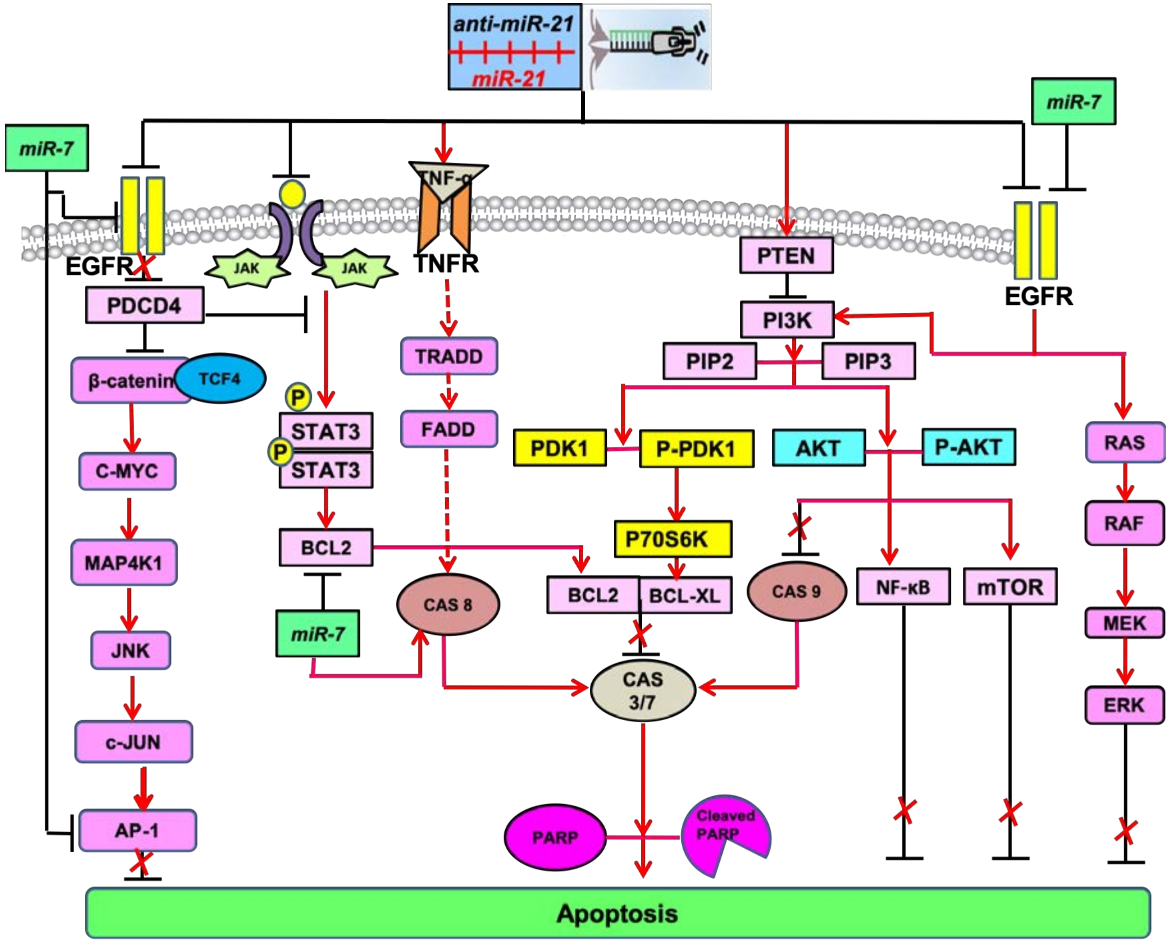

Suppl. Figure 7

Uncropped Western Blots for Fig. 2C

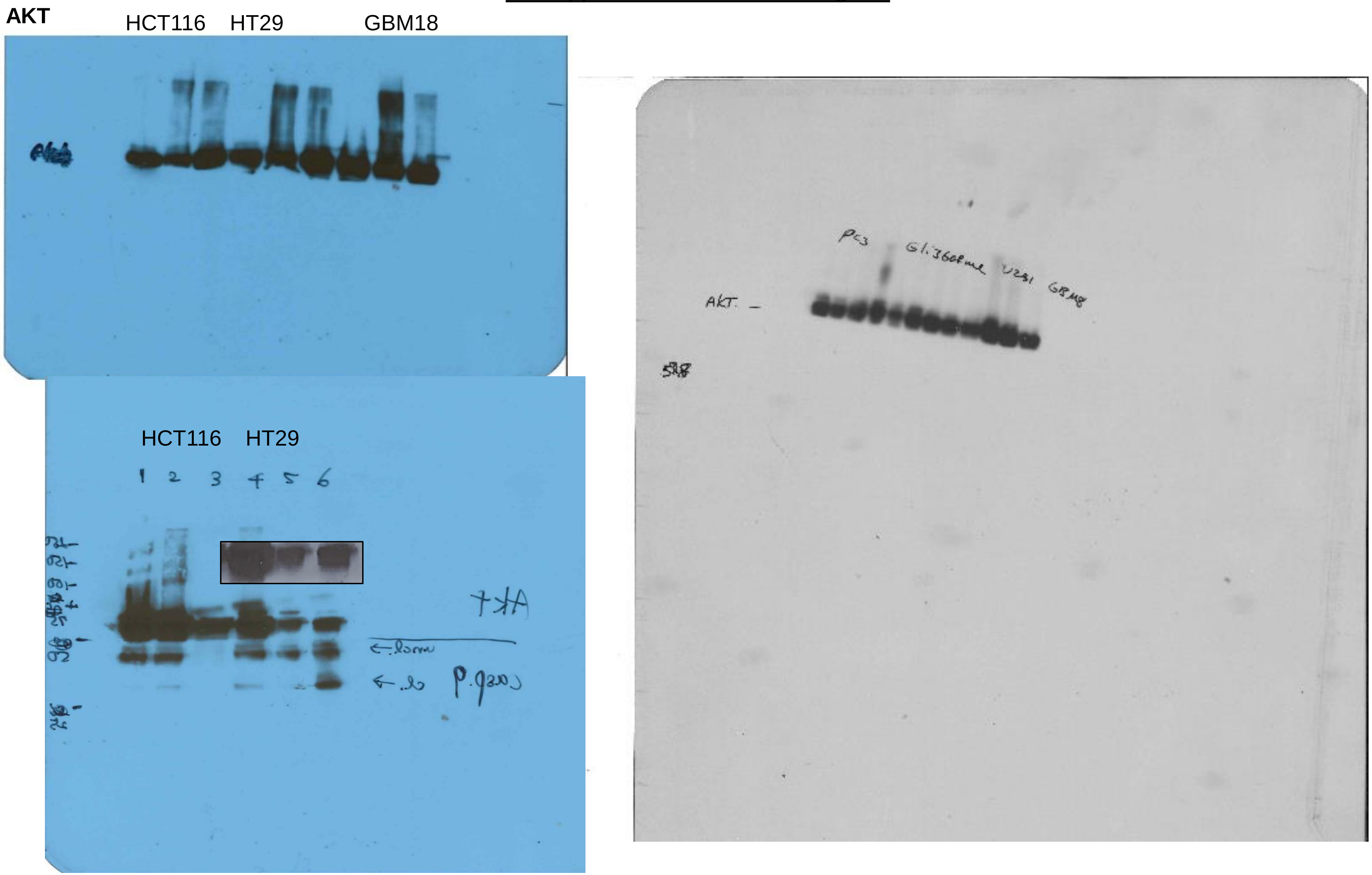

Uncropped Western Blots for Fig. 2C

P-AKT

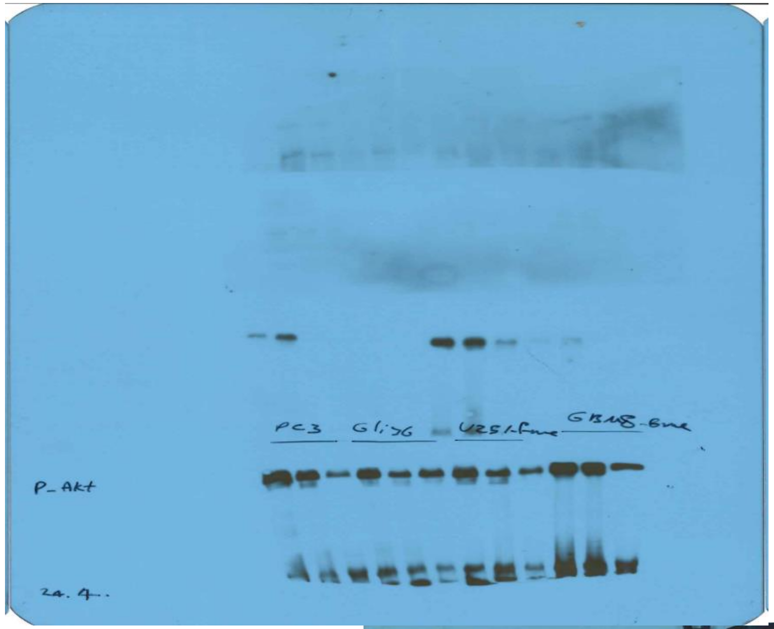

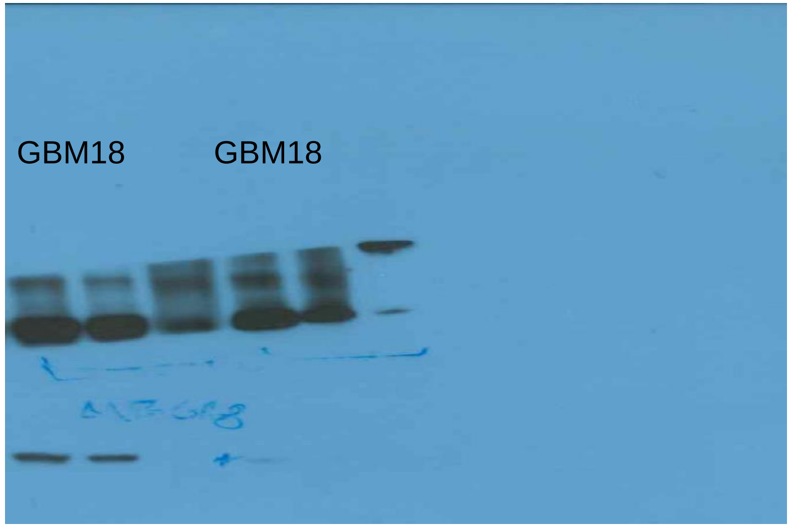

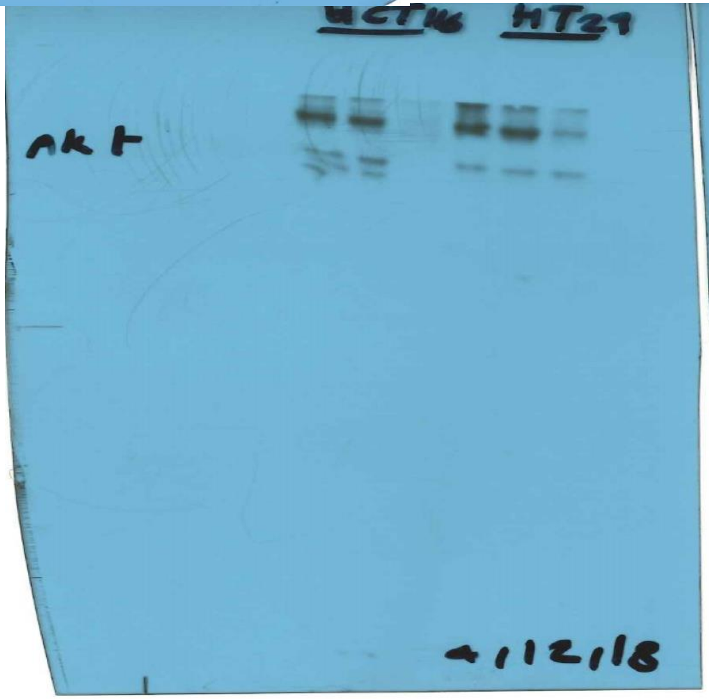

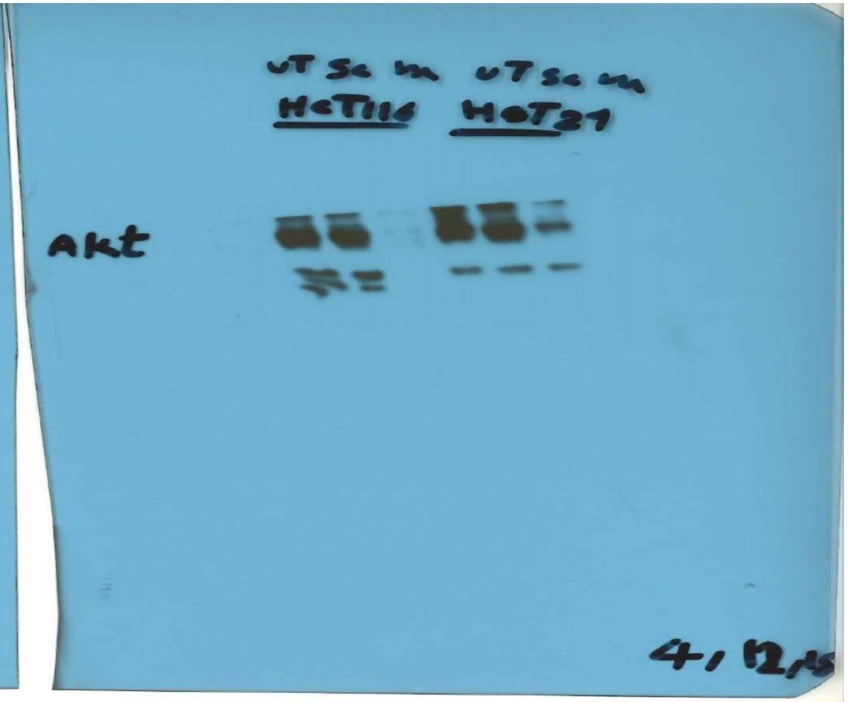

Uncropped Western Blots for Fig. 2C

PTEN

Gli36

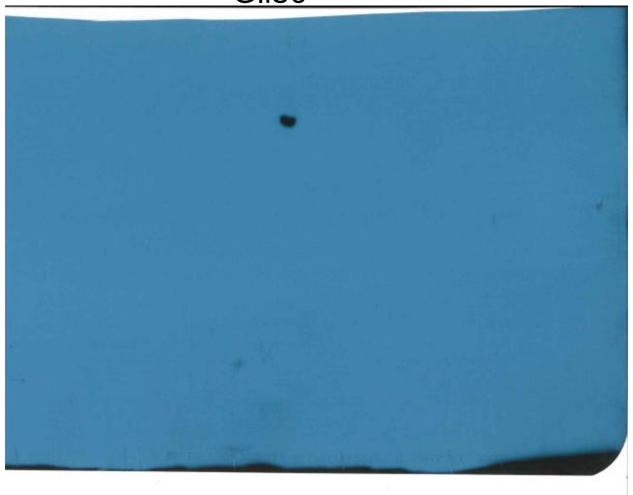

GBM8

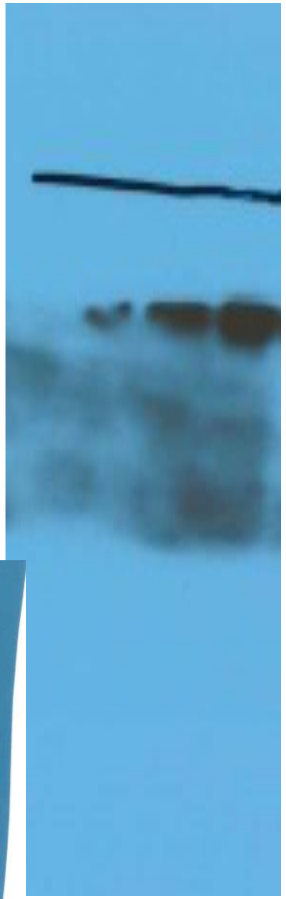

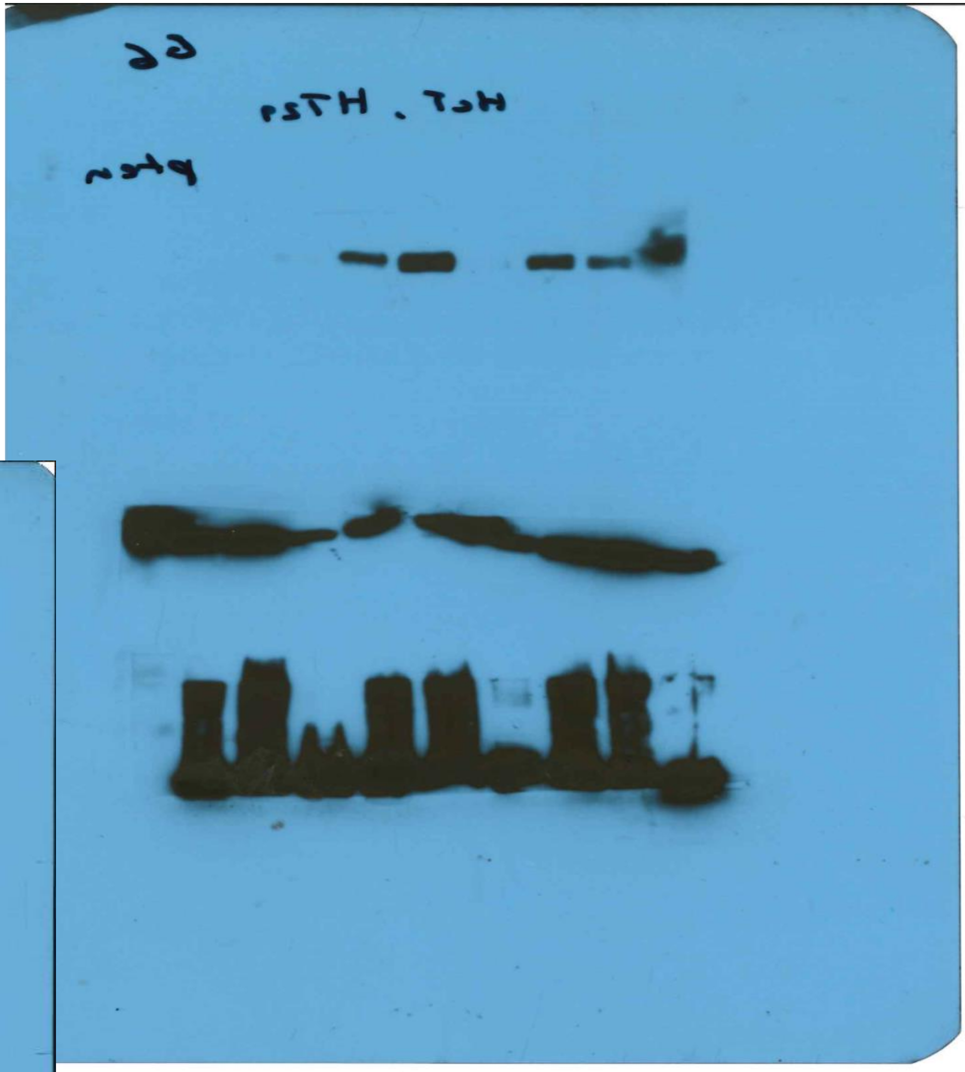

PC3

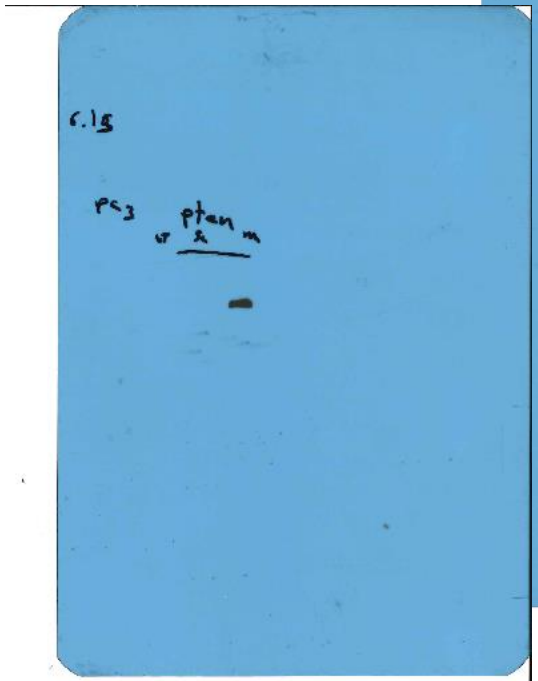

GBM18

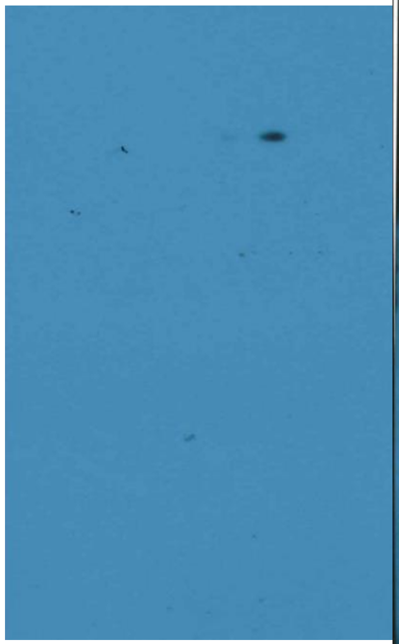

U251

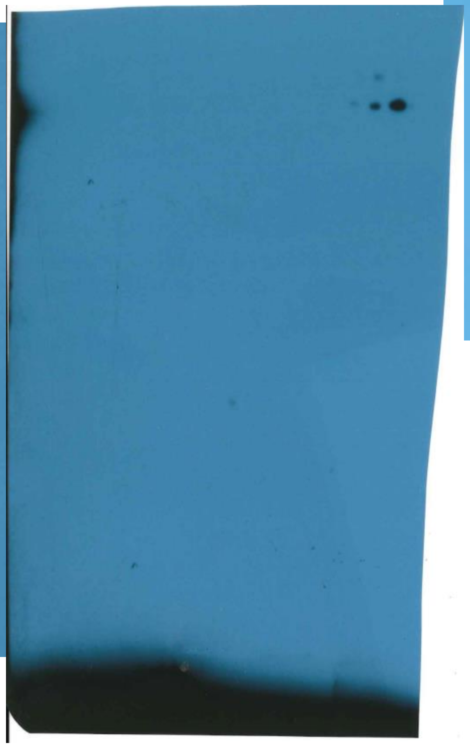

Uncropped Western Blots for Fig. 2C

Cl. PARP

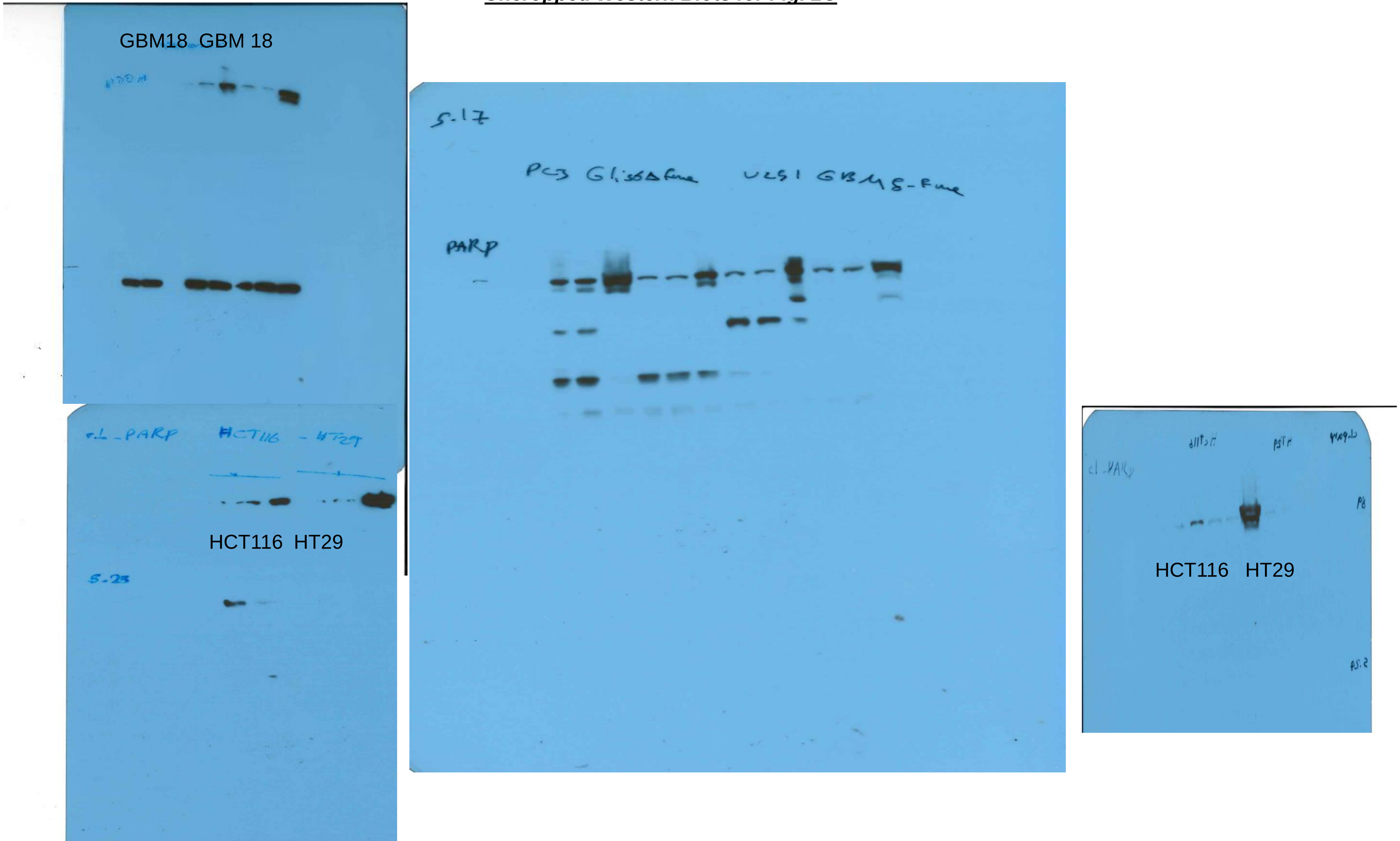

Uncropped Western Blots for Fig. 2C

GBM18

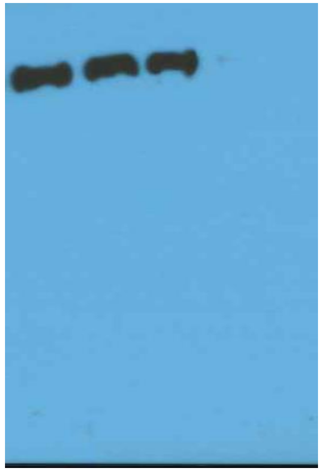

Tubulin

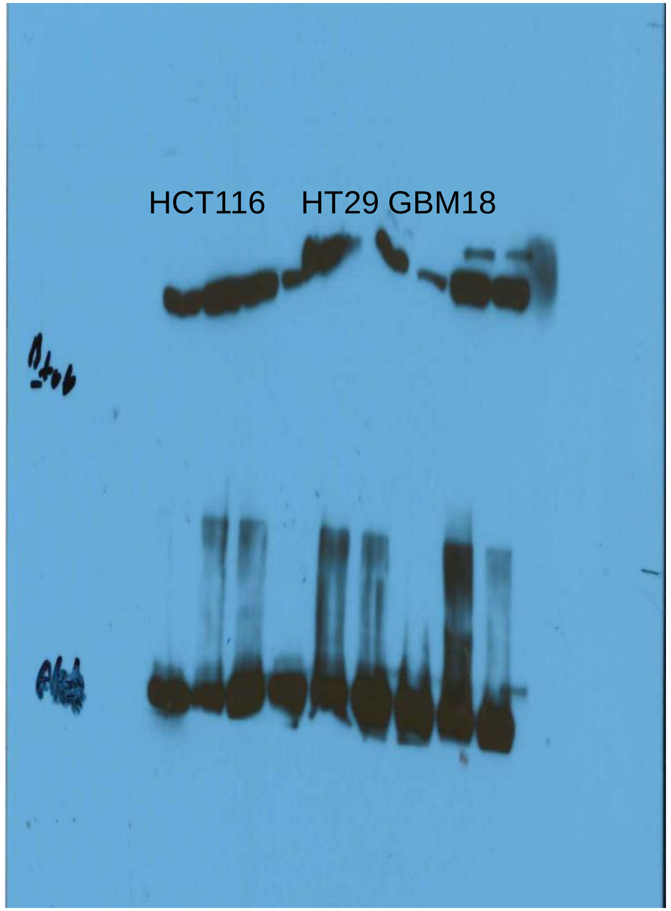

HT29

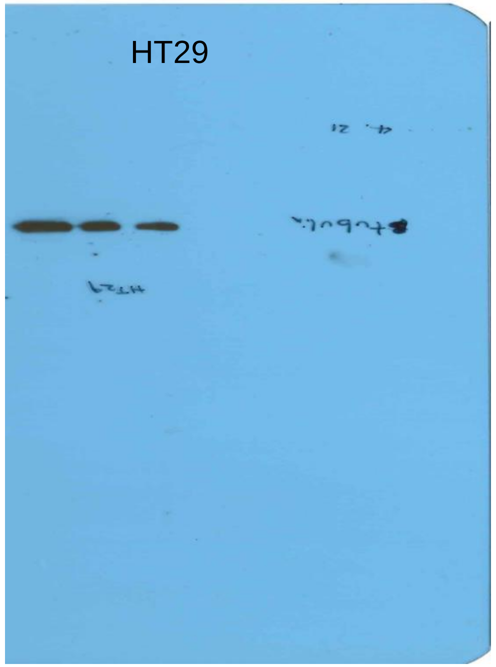

PC3 GLi36 U251 GBM8

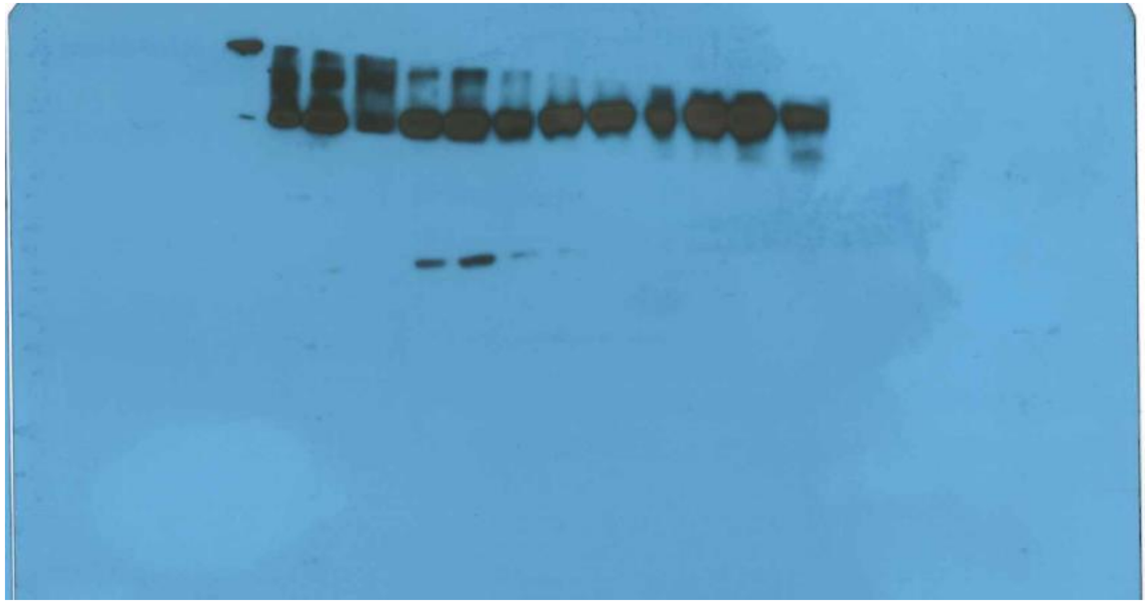

Uncropped Western Blots for Fig. 4B

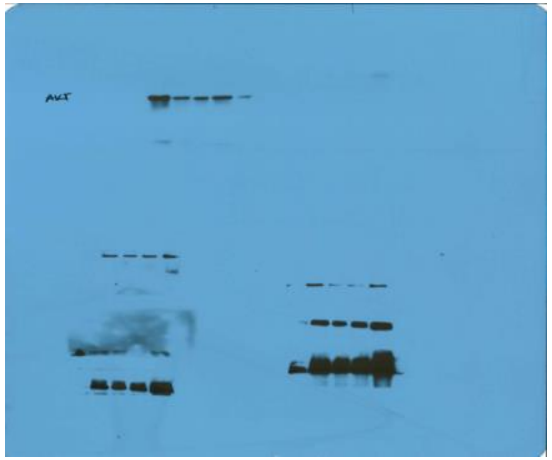

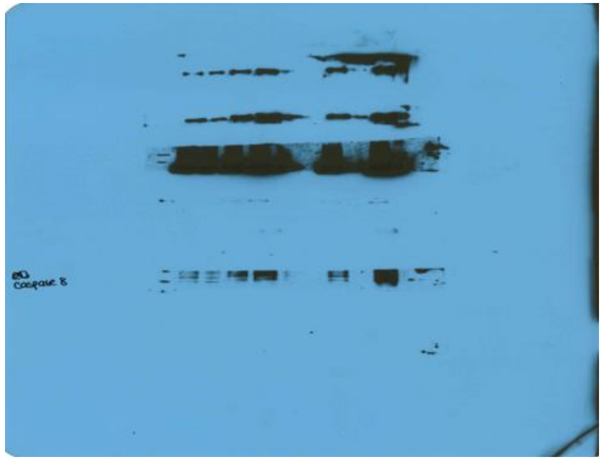

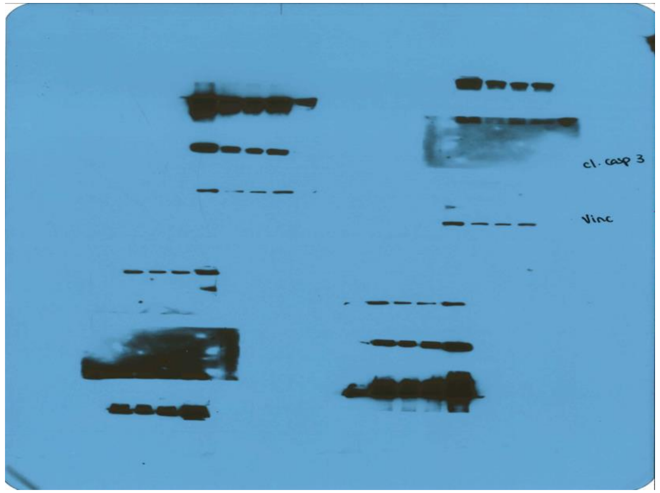

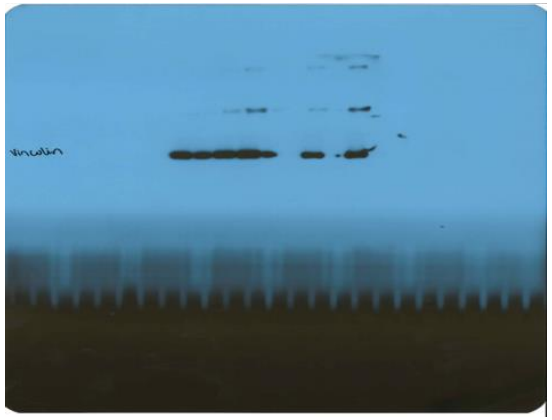

Supplement: Supplementary file 2 — Supplementary Figures. [file 41598_2024_66236_MOESM2_ESM.pdf]
